# Inter- and intra-species heterogeneity in germination of *Aspergillus* conidia

**DOI:** 10.1007/s10482-022-01762-4

**Published:** 2022-07-20

**Authors:** Maryam Ijadpanahsaravi, Wieke R. Teertstra, Han A. B. Wösten

**Affiliations:** Microbiology, Department of Biology, Utrecht University, Padualaan 8, 3584 CH Utrecht, The Netherlands

**Keywords:** Fungus, *Aspergillus*, Conidia, Swelling, Germination, Amino acids

## Abstract

Aspergilli are among the most abundant fungi worldwide. They degrade organic material and can be pathogens of plants and animals. Aspergilli spread by forming high numbers of conidia. Germination of these stress resistant asexual spores is characterized by a swelling and a germ tube stage. Here, we show that conidia of *Aspergillus* *niger,* *Aspergillus* *oryzae,* *Aspergillus* *clavatus*, *Aspergillus* *nidulans* and *Aspergillus* *terreus* show different swelling and germ tube formation dynamics in pure water or in water supplemented with (in)organic nutrients. Apart from inter-species heterogeneity, intra-species heterogeneity was observed within spore populations of the aspergilli except for *A.* *terreus*. Sub-populations of conidia differing in size and/or contrast showed different swelling and germ tube formation dynamics. Together, data imply that aspergilli differ in their competitive potential depending on the substrate. Moreover, results suggest that intra-species heterogeneity provides a bet hedging mechanism to optimize survival of aspergilli.

**Supplementary Information:**

The online version contains supplementary material available at 10.1007/s10482-022-01762-4.

## Introduction

Aspergilli are among the most abundant fungi worldwide. They have a saprotrophic life style and, as such, are also important food and feed spoilers (Magan and Aldred 2007). Moreover, aspergilli can be opportunistic pathogens of plants and animals. On the other hand, aspergilli are important cell factories for the production of enzymes and small molecules such as organic acids (Wösten 2019). The success of aspergilli is explained by dispersal of high numbers of asexual spores, called conidia, and the fact that they can grow in a wide range of environmental conditions (Bennett 2010; Krijgsheld et al. 2013). The latter is facilitated by the capacity of *Aspergillus* conidia to change transcription in response to the environment until these spores leave the conidiophore. This transcriptional response prepares conidia to grow under environmental conditions during which the spores had been formed (Wang et al. 2021).

Conidia are spread by wind, water, and other vectors such as animals. These spores are resistant to stresses such as drought, UV, reactive oxygen species and heat (Wyatt et al. 2013; Dijksterhuis 2019). In contrast, vegetative hyphae are stress sensitive. Thus, the fungus switches from a stress-resistant to a stress-sensitive state upon germination of conidia. Germination of conidia is described as a two-step process*.* First, the spore is swelling. This isotropic phase is followed by polarized growth during which a germ tube is formed. It should be noted that germ tube formation can also occur without detectable swelling (Punt et al 2022).

Environmental factors such as water availability, temperature, and nutrients impact germination of *Aspergillus* (Hayer et al. 2013, 2014; Ijadpanahsaravi et al. 2021; Marin et al. 1998; Osherov and May 2001). For instance, the estimated maximum number of spores (*P*_max_) that are activated to swell and to form germ tubes is < 1% when conidia of *A.* *niger* are exposed to water or 50 mM glucose (Ijadpanahsaravi et al. 2021). Combining glucose with either NaNO_3_, KH_2_PO_4_, or MgSO_4_ increases incidence of swelling and germination of these spores with a *P*_max_ up to 15% and 5.4%, respectively, in a 16 h time frame. Swelling and germ tube formation is further increased up to 26% and 11% by mixing glucose with a combination of these inorganic components. High and intermediate inducing amino acids can replace glucose to initiate germination (Ijadpanahsaravi et al. 2021). For instance, the high inducing amino acid proline results in a *P*_max_ of swelling and germ tube formation of 97% and 55%, respectively. Together, a combination of an inducing carbon source with either inorganic phosphate, inorganic nitrogen or magnesium sulphate is the minimum requirement for *A.* *niger* conidia to germinate. Here we studied the effect of inorganic nutrients and amino acids on germination of (sub-)populations of conidia of cultures of *A.* *niger,* *Aspergillus* *clavatus,* *Aspergillus* *nidulans,* *Aspergillus* *oryzae* and *Aspergillus* *terreus.* These aspergilli are shown to differ in their potential to germinate in pure water or water containing (in)organic nutrients. Moreover, it is shown that germination dynamics of sub-populations of large and small conidia of aspergilli or sub-populations with high or low contrast can be different. Data imply that aspergilli have different competitive potential in different substrates and use a bed hedging mechanism in their germination response.

## Material and methods

### Strains and culture conditions

*A.* *niger* N402 (Bos et al. 1988), *A.* *oryzae* RIB40 (Machida et al. 2005), *A.* *clavatus* NRRL1 (Arnaud et al. 2012), *A.* *nidulans* FGSC A4 (Arnaud et al. 2012) and *A.* *terreus* NHI 2624 (Arnaud et al. 2012) were routinely grown at 30 °C on minimal medium (MM) with 1% glucose and 1.5% agar as described (Ijadpanahsaravi et al. 2021). Spores were harvested with a cotton swab wetted with water from 7 day-old-colonies, taken up in water and filtered through cotton wool to remove hyphae. Spores were washed 2 times with water with intermittent centrifugation at 4000 g for 5 min at 4 °C and kept at this temperature up to the moment of inoculation. Spores were counted with a Buerker Tuerk counter and diluted to a final density of 2.7 10^5^ ml^−1^. Wells of a 96 well suspension culture plate (Greiner bio-one, Cellstar 655,185, www.gbo.com) were inoculated with 15 µl of this suspension (i.e. 40,000 spores per well).

### Germination analysis

Swelling of conidia and germ tube formation was monitored for 24 h at 30 °C using an oCelloScope (Biosense Solutions, www.biosensesolutions.dk) (Fredborg et al. 2013) with UniExplorer software version 8.1.0.7682-RL2 and the X–Y segmentation plug in 6.0.0.811. Swelling and germ tube formation was monitored in water, 10 mM glucose, 2 mM MgSO_4_, 25 mM NaPO_4_ buffer (pH 6), 10 mM NaNO_3_ or mixtures thereof. In addition, swelling and germ tube formation was monitored in 25 mM NaPO_4_ buffer, 2 mM MgSO_4_ and 10 mM of one out of the 20 proteinogenic L- amino acids (pH was 6 in all cases except for tyrosine that resulted in a pH of 2). As a control, conidia were monitored that had been inactivated at 70 °C for 15 min. Measurements (using at least biological triplicates) were started after 1 h of incubation to enable settling of the conidia at the bottom of the well. Objects were scanned every hour during the first 10 h and every 2 h during the next 14 h. The scan area length was set at 405 µm, the object area (min–max) at 70–700 pixels (with pixels of 0.55 × 0.55 µm) and the maximum number of objects at 1000. Features were Area, Circularity and X and Y coordinates when whole spore populations were analyzed, while contrast was added in the case subpopulations were studied (see below). Data was used as input in an asymmetric model (Dantigny et al. 2011) in the R package GrowthRates (Petzoldt 2019) and using the Levenberg–Marquardt algorithm (Ijadpanahsaravi et al. 2021). *P*_max_, τ, and d are the output of the model. *P*_max_ is the maximal percentage of swollen conidia or conidia forming germ tubes, while τ (h) is the time where *P* = 0.5 *P*_max_ and d represents the degree of heterogeneity in the response of conidia. In most of the inducing conditions, too many objects had formed hyphae after 15 h that could not be traced back to one of the objects or that obscured other objects. Therefore, data from t = 1 h to t = 15 h were used for modelling as described (Ijadpanahsaravi et al. 2021). Parameters were limited to *P* ≥ 0 and ≤ 120%, τ ≥ 1 and ≤ 15, d ≥ 2 and ≤ 30 when fitting the model. The τ ≥ 1 and ≤ 15 corresponded to the data that were used for modelling (see above), while d ≥ 2 and ≤ 30 was used to ensure a S-curve as output of the model. Objects were classified as resting conidia, swollen conidia, and conidia forming germ tubes based on their surface area and circularity (Table 1).

**Table 1:** Classification of resting conidia, swollen conidia, and conidia forming germ tubes in pure cultures based on circularity (C) and surface area (SA) in pixels (p)

	Resting	swollen	Germ tube	Threshold
*A.* *niger* total & low and high contrast	SA ≤ 150 p C > 0.97	SA > 150 p C > 0.97	SA > 150 p C ≤ 0.97	SA > 300 p
*A.* *niger* small size	SA ≤ 135 p C > 0.97	SA > 135 p C > 0.97	SA > 135 p C ≤ 0.97	SA > 270 p
*A.* *niger* large size	SA ≤ 160 p C > 0.97	SA > 160 p C > 0.97	SA > 160p C ≤ 0.97	SA > 320 p
*A.* *clavatus* total & low and high contrast	SA ≤ 150 p C > 0.97	SA > 150 p C > 0.97	SA > 150 p C ≤ 0.97	SA > 300 p
*A.* *clavatus* small size	SA ≤ 120 p C > 0.97	SA > 120 p C > 0.97	SA > 120 p C ≤ 0.97	SA > 240 p
*A.* *clavatus* large size	SA ≤ 145 p C > 0.97	SA > 145 p C > 0.97	SA > 145 p C ≤ 0.97	SA > 290 p
*A.* *nidulans* total & low and high contrast	SA ≤ 125 p C > 0.97	SA > 125 p C > 0.97	SA > 125 p C ≤ 0.97	SA > 250 p
*A.* *nidulans* small size	SA ≤ 105 p C > 0.97	SA > 105 p C > 0.97	SA > 105 p C ≤ 0.97	SA > 210 p
*A.* *nidulans* large size	SA ≤ 120 p C > 0.97	SA > 120 p C > 0.97	SA > 120 p C ≤ 0.97	SA > 240 p
*A.* *oryzae* total & low and high contrast	SA ≤ 225 p C > 0.97	SA > 225 p C > 0.97	SA > 225 p C ≤ 0.97	SA > 450 p
*A.* *oryzae* small size	SA ≤ 150 p C > 0.97	SA > 150 p C > 0.97	SA > 150 p C ≤ 0.97	SA > 300 p
*A.* *oryzae* large size	SA ≤ 190 p C > 0.97	SA > 190 p C > 0.97	SA > 190 p C ≤ 0.97	SA > 380 p
*A.* *terreus* total & low and high contrast	SA ≤ 100 p C > 0.97	SA > 100 p C > 0.97	SA > 100 p C ≤ 0.97	SA > 200 p
*A.* *terreus* small size	SA ≤ 75 p C > 0.97	SA > 75 p C > 0.97	SA > 75 p C ≤ 0.97	SA > 150 p
*A.* *terreus* large size	SA ≤ 85 p C > 0.97	SA > 85 p C > 0.97	SA > 85 p C ≤ 0.97	SA > 170 p

Objects were removed from the data set from the time point they had reached the SA threshold

Size and contrast were used to define sub-populations within the total spore population of each of the aspergilli. The germination responses of these sub-populations was studied. To this end, the 5% smallest and largest conidia or the 5% spores with the highest and lowest contrast were removed from each data set, respectively, to avoid outliers impacting the germination analysis. Germination dynamics was assessed with the resulting 15% smallest and largest conidia and with the resulting 15% conidia with the highest and lowest contrast. Area and circularity criteria were the same for the total population of conidia and for the populations of low and high contrast to analyse *P*_max_, and d of swellling and germination in the asymmetric model. However, criteria were adapted to be able to analyze the large and small subpopulations of the spores (Table 1). The area that was selected to classify a spore as swelling was ≥ 10 pixels of the area of the largest conidium in the sub-population.

The R package ‘pheatmap’ was used for hierarchical clustering of the swelling and germination responses of the spores of the aspergilli in the different media. Normalization was done using *P*_max_ (x) − *P*_max_(PS)/(100 − *P*_max_(PS)*100.

## Results

### Effect of nitrate, phosphate, sulphate and glucose on germination

Light microscopy and oCelloScope imaging was used to monitor swelling and germ tube formation of conidia of 7 day-old cultures of *A.* *clavatus, A. nidulans, A. oryzae, A. terreus,* and *A. niger*. To this end, conidia of these species were incubated in Milli-Q water either or not in the presence of 25 mM Na-phosphate buffer (P), 2 mM MgSO_4_ (S), 10 mM NaNO_3_ (N), or 10 mM glucose (G), or combinations thereof. Spores of the tested aspergilli did not settle at the bottom of the 96 wells plate when inoculated in water or water containing glucose. This disabled automated oCelloScope imaging and, therefore, swelling and germ tube formation could only be followed with light microscopy. No germination of *A. niger* and *A. terreus* conidia was observed in pure water even after 72 h incubation (Suppl. Figure 1). In contrast, about 1% of the conidia of *A. oryzae* had germinated in a 24 h period, while 25% and 40% of the *A. clavatus* and *A. nidulans* spores had formed germ tubes, respectively. Germination incidence increased for all aspergilli when glucose was added to water; ≤ 2% of the *A. niger* and *A. terreus* spores had formed germ tubes in the 24 h period*,* while this was 10%, 30% and 50% in the case of *A. oryzae, A. clavatus* and *A. nidulans*, respectively (data not shown). These data show that aspergilli differ in their potential to germinate in pure water and that glucose increases germination incidence in the absence of inorganic nutrients.

Next, swelling of conidia and germ tube formation was studied with oCelloScope imaging in water containing N, P, or S in presence or absence of glucose and combinations thereof. Data was analysed using an asymmetric model (Dantigny et al. 2011). The outcomes of this model (*P*_max_, τ, and d) describe the process of swelling and germ tube formation. *P*_max_ represents the maximal percentage of swollen spores or conidia forming germ tubes, τ represents the time when *P* = 0.5 *P*_max,_ and d represents the heterogeneity within the spore population. Here, we focus on *P*_max_ for readability. No swelling (Table 2) and germination (Table 3) of *A. niger* conidia was observed when inoculated in N, P or S, while ≤ 8.25% of the spores germinated when mixing two or three of these components. N (*P*_max_ swelling and germination 4.54% and 0.4%, respectively), but not P or S, induced swelling (Table 2) and germination (Table 3) in the case of *A. terreus*, while combinations of two or three of these components resulted in a *P*_max_ of swelling ≤ 12.66% and a *P*_max_ of germination ≤ 12.52%. Presence of N, P, or S or their combinations hardly, if at all, increased germination of *A. clavatus* (*P*_max_ ≤ 19.50%), *A. oryzae* (*P*_max_ ≤ 15.75%) and *A. nidulans* (*P*_max_ ≤ 33.37%) conidia when compared to pure water, while *P*_max_ of swelling was ≤ 27.97%, ≤ 28.53, and ≤ 53.53%, respectively. Together, data show that N, P, and S have a minor effect on germination in all tested aspergilli. Adding glucose to the media hardly, if at all, increased the incidence of swelling (*P*_max_ ≤ 8.51%) and germination (*P*_max_ ≤ 7.53%) in the case of *A. terreus* but did increase swelling and germination incidence in the other aspergilli. *P*_max_ of swelling was ≤ 48.97%, ≤ 97.43%, ≤ 76.62%, and ≤ 93.95% in the case of *A. niger, A. clavatus, A. oryzae* and *A. nidulans*, respectively, while *P*_max_ of germination corresponded to ≤ 32.78, ≤ 91.02%, ≤ 54.86%, and ≤ 85.46% (Tables 2, 3).

**Table 2:** Parameter estimates of the asymmetrical model describing swelling of conidia in Milli-Q water either or not supplemented with 10 mM glucose (G), 10 mM NaNO_3_ (N), 25 mM NaPO_4_ buffer pH 6.0 (P), 2 mM MgSO_4_ (S) or combinations thereof

Component	*P*_max_ (%)	(h)	d (−)	RMSE	N	M
*A.* *niger*						
N	1.00 [0.54;1.46]	15.00 [11.77;18.22]	30.0 [− 64.82;124.82]	0.11	269	8
P	1.36 [− 0.71;3.43]	5.72 [− 10.64;22.08]	1.36 [− 2.54;5.25]	0.14	623	6
S	2.74 [− 1.36;6.84]	8.24 [− 19.72;36.19]	1.00 [− 0.69;2.69]	0.11	410	14
GN	32.15 [30.08;34.22]	2.93 [2.51;3.36]	1.90 [1.36;2.44]	0.43	677	221
GP	26.00 [20.89;31.11]	4.72 [3.2;6.23]	2.46 [0.51;4.42]	1.02	668	10
GS	9.06 [7.24;10.89]	5.65 [3.88;7.42]	1.97 [0.88;3.06]	0.22	785	10
NP	8.25 [− 0.05;16.55]	15.00 [− 4.69;34.69]	1.70 [0.33;3.06]	0.17	424	12
NS	0.96 [0.65;1.28]	1.11 [− 0.44;2.66]	1.51 [− 2.1;5.11]	0.07	652	12
PS	1.01 [− 1.18;3.21]	6.58 [− 26.37;39.53]	1.00 [− 1.9;3.9]	0.08	736	5
GNP	30.72 [26.07;35.37]	4.86 [3.7;6.01]	3.58 [0.67;6.5]	1.29	693	38
GNS	18.99 [8.7;29.28]	5.32 [0.54;10.09]	1.74 [− 0.67;4.15]	1.12	666	14
GPS	19.75 [17.63;21.87]	4.43 [3.62;5.24]	3.36 [1.4;5.32]	0.59	708	19
NPS	1.16 [0.81;1.5]	2.02 [0.43;3.6]	1.44 [− 0.43;3.31]	0.06	697	11
GNPS	48.97 [42.36;55.58]	4.86 [3.82;5.91]	2.91 [1.11;4.71]	1.55	618	226
*A.* *terreus*						
N	4.58 [3.85;5.32]	1.00 [0.39;1.61]	3.73 [− 9.02;16.48]	0.31	433	0
P	2.87 [− 3.39;9.13]	15.00 [− 52.96;82.96]	1.00 [− 0.53;2.53]	0.09	940	8
S	0.87 [0.18;1.56]	1.32 [− 2.36;5]	1.41 [− 5.18;8.00]	0.14	193	4
GN	7.48 [− 0.69;15.66]	3.09 [− 4.89;11.07]	1.00 [− 1.51;3.51]	0.56	976	38
GP	4.95 [2.72;7.17]	2.22 [− 0.16;4.6]	1.00 [− 0.29;2.29]	0.19	999	25
GS	3.19 [1.17;5.2]	1.00 [− 1.04;3.04]	1.00 [− 2.21;4.21]	0.24	669	6
NP	2.89 [− 3.6;9.38]	15.00 [− 23.11;53.11]	2.01 [− 1.93;5.96]	0.16	622	11
NS	3.49 [2.23;4.75]	1.00 [− 0.17;2.17]	1.00 [− 0.83;2.83]	0.15	752	6
PS	3.84 [3.02;4.66]	1.00 [0.06;1.94]	3.08 [− 7.91;14.08]	0.34	853	4
GNP	5.79 [3.8;7.78]	1.00 [− 0.11;2.11]	1.00 [-0.74;2.74]	0.24	565	5
GNS	3.79 [2.91;4.67]	1.00 [0.25;1.75]	1.00 [− 0.18;2.18]	0.11	879	5
GPS	5.1 [2.98;7.21]	2.28 [0.02;4.54]	1.00 [− 0.17;2.17]	0.17	775	20
NPS	12.66 [− 8.28;33.61]	15.00 [− 21.73;51.73]	1.46 [− 0.37;3.3]	0.39	863	4
GNPS	8.51 [0.22;16.8]	11.59 [− 1.73;24.92]	2.18 [− 0.61;4.97]	0.39	587	8
*A.* *oryzae*						
N	3.76 [− 1.58;9.1]	15.00 [− 7.51;37.51]	2.20 [− 0.67;5.06]	0.15	404	9
P	6.45 [0.91;11.98]	15.00 [− 3.91;33.91]	1.48 [0.51;2.44]	0.10	486	17
S	16.48 [6.32;26.63]	15.00 [5.14;24.86]	2.17 [0.95;3.39]	0.28	348	14
GN	62.74 [− 8.58;134.06]	14.1 [− 9.37;37.57]	1.51 [0.1;2.92]	1.53	370	24
GP	50.75 [23.55;77.96]	10.38 [1.66;19.09]	1.53 [0.57;2.48]	0.98	409	11
GS	23.09 [19.17;27.02]	6.44 [4.89;7.99]	2.24 [1.18;3.29]	0.50	405	40
NP	14.1 [9.17;19.03]	15.00 [12.32;17.68]	6.94 [− 0.05;13.93]	0.53	447	12
NS	14.29 [0.16;28.43]	15.00 [4.16;25.84]	3.57 [− 1.34;8.49]	0.67	219	7
PS	12.59 [10.31;14.87]	15.00 [13.47;16.53]	5.43 [3.27;7.6]	0.18	547	13
GNP	73.15 [65.87;80.42]	7.86 [6.92;8.81]	3.00 [2.1;3.9]	1.05	461	25
GNS	58.43 [− 38.59;155.46]	15.00 [− 21.57;51.57]	1.48 [− 0.39;3.34]	1.82	392	31
GPS	47.71 [30.46;64.97]	7.12 [3.46;10.79]	2.05 [0.36;3.74]	1.68	470	21
NPS	28.53 [18.84;38.21]	14.84 [12.26;17.42]	7.27 [− 0.37;14.91]	1.14	464	9
GNPS	76.62 [71.08;82.17]	6.71 [6.13;7.29]	4.59 [2.83;6.36]	1.45	513	119
*A.* *clavatus*						
N	7.62 [− 13.08;28.31]	15.00 [− 64.77;94.77]	1.07 [− 0.98;3.11]	0.30	489	11
P	19.33 [11.54;27.13]	12.29 [7.75;16.83]	3.12 [0.99;5.25]	0.53	613	12
S	19.51 [16.2;22.81]	14.27 [12.88;15.65]	6.00 [3.2;8.81]	0.35	406	13
GN	90.76 [76.91;104.61]	7.96 [6.35;9.56]	2.24 [1.49;2.99]	1.30	515	59
GP	79.57 [61.94;97.2]	6.41 [4.29;8.52]	1.98 [0.91;3.05]	1.86	696	12
GS	66.01 [25.99;106.02]	7.13 [− 0.4;14.67]	1.45 [0.01;2.89]	2.29	477	9
NP	16.96 [10.51;23.42]	13.2 [9.83;16.56]	5.14 [− 0.1;10.37]	0.70	417	9
NS	5.55 [3.67;7.44]	2.29 [0.44;4.15]	1.00 [0.05;1.95]	0.15	603	3
PS	22.58 [19.09;26.07]	13.82 [12.26;15.38]	3.93 [2.84;5.03]	0.24	658	11
GNP	92.45 [90.58;94.33]	5.08 [4.95;5.21]	6.73 [5.7;7.76]	0.64	362	14
GNS	90.87 [73.48;108.25]	7.88 [5.99;9.77]	2.59 [1.32;3.86]	2.05	668	6
GPS	84.29 [67.82;100.76]	6.91 [4.75;9.08]	1.66 [1.04;2.28]	1.21	646	7
NPS	27.97 [14.26;41.67]	15.00 [10.28;19.72]	4.32 [0.71;7.93]	0.81	617	25
GNPS	97.43 [95.27;99.59]	4.24 [4.1;4.38]	6.40 [5.21;7.6]	0.78	526	13
*A.* *nidulans*						
N	28.29 [15.53;41.05]	15.00 [11.34;18.66]	5.89 [− 0.5;12.28]	1.11	338	10
P	53.52 [41.54;65.51]	15.00 [13.14;16.86]	5.62 [2.75;8.5]	0.98	348	12
S	16.79 [12.09;21.49]	15.00 [12.84;17.16]	6.74 [1.48;12.01]	0.48	230	27
GN	82.91 [70.13;95.69]	9.32 [7.74;10.89]	3.10 [1.91;4.3]	1.50	515	105
GP	91.55 [83.85;99.25]	9.94 [9.11;10.78]	3.81 [2.9;4.71]	1.06	481	36
GS	25.28 [10.38;40.18]	15.00 [4.6;25.4]	1.92 [0.96;2.88]	0.36	548	86
NP	23.08 [10.49;35.68]	14.99 [10.88;19.1]	7.37 [− 4.97;19.71]	1.45	206	27
NS	13.93 [− 46.72;74.58]	15.00 [− 99.07;129.07]	1.21 [− 2.6;5.02]	0.95	422	10
PS	51.52 [42.96;60.08]	15.00 [13.58;16.42]	5.26 [3.41;7.12]	0.65	638	29
GNP	87.23 [80.53;93.92]	7.01 [6.37;7.65]	4.36 [2.72;6.01]	1.63	414	52
GNS	74.89 [47.89;101.89]	15.00 [9.63;20.37]	2.37 [1.55;3.2]	0.80	505	34
GPS	85.52 [80.31;90.73]	10.65 [10.06;11.24]	4.31 [3.53;5.09]	0.74	442	35
NPS	48.68 [37.57;59.79]	15.00 [13.29;16.71]	7.60 [2.11;13.08]	1.34	649	34
GNPS	93.95 [88.3;99.59]	8.03 [7.5;8.56]	5.09 [3.6;6.58]	1.34	328	27

Confidence intervals are indicated between brackets, N represents the number of objects at t = 1 h, while M represents the number of objects that could no longer be monitored between 2 and 16 h because the hypha had become too long or the object was obscured by hyphae of other objects. RMSE represents the root mean square error of the modelled data

**Table 3:** Parameter estimates of the asymmetrical model describing germ tube formation in Milli-Q water either or not supplemented with 10 mM glucose (G), 10 mM NaNO_3_ (N), 25 mM NaPO_4_ buffer pH 6.0 (P), 2 mM MgSO_4_ (S) or combinations thereof

Component	*P*_max_ (%)	(h)	d (−)	RMSE	N	M
*A.niger*						
N	0.4 [0.2;0.6]	15.00 [11.54;18.46]	30.0 [− 71.74;131.74]	0.05	269	8
P	0.53 [0.26;0.8]	5.89 [2.11;9.68]	5.08 [− 10.61;20.77]	0.08	623	6
S	0.84 [− 0.12;1.8]	15.00 [1.82;28.18]	3.31 [− 1.55;8.18]	0.04	410	14
GN	17.36 [11.51;23.21]	3.52 [1.16;5.87]	1.54 [− 0.1;3.19]	0.78	677	221
GP	1.01 [− 0.15;2.16]	12.24 [-3.38;27.85]	2.30 [− 1.05;5.64]	0.05	668	10
GS	2.13 [− 3.84;8.09]	13.8 [− 47.09;74.69]	1.40 [− 1.81;4.6]	0.12	785	10
NP	4.27 [1.28;7.27]	15 [8.33;21.67]	4.40 [− 0.97;9.77]	0.18	424	12
NS	0.45 [0.06;0.85]	1.62 [− 1.9;5.15]	1.00 [− 2.11;4.11]	0.04	652	12
PS	0.40 [− 0.05;0.85]	1.31 [− 2.59;5.22]	1.00 [− 3.62;5.62]	0.05	736	5
GNP	15.71 [− 1.93;33.36]	15.00 [− 6.89;36.89]	1.70 [0.17;3.24]	0.38	693	38
GNS	6.52 [1.21;11.84]	10.78 [− 1.52;23.08]	1.76 [0.01;3.51]	0.22	666	14
GPS	3.20 [− 4.94;11.34]	15.00 [− 41.09;71.09]	1.47 [− 1.38;4.32]	0.15	708	19
NPS	0.58 [0.05;1.1]	3.30 [− 2.9;9.5]	1.44 [− 2.63;5.52]	0.07	697	11
GNPS	32.78 [25.3;40.27]	9.64 [7.28;12]	3.12 [1.41;4.83]	0.83	618	226
*A.terreus*						
N	4.40 [3.56;5.24]	1.00 [0.06;1.94]	2.53 [− 4.06;9.12]	0.32	433	0
P	2.60 [− 2.46;7.66]	15.00 [− 45.64;75.64]	1.00 [− 0.36;2.36]	0.07	940	8
S	0.87 [0.18;1.56]	1.32 [− 2.36;5]	1.41 [− 5.18;8]	0.14	193	4
GN	6.54 [− 0.98;14.06]	4.10 [− 7.03;15.22]	1.00 [− 1.17;3.17]	0.41	976	38
GP	4.72 [1.91;7.52]	2.88 [− 1.18;6.93]	1.00 [− 0.43;2.43]	0.20	999	25
GS	2.43 [1;3.85]	1.00 [− 0.89;2.89]	1.00 [− 1.97;3.97]	0.17	669	6
NP	2.85 [− 2.42;8.12]	15.00 [− 12.69;42.69]	2.36 [− 1.85;6.57]	0.16	622	11
NS	3.12 [1.8;4.44]	1.00 [− 0.37;2.37]	1.00 [− 1.15;3.15]	0.16	752	6
PS	4.14 [1.33;6.95]	1.00 [− 1.19;3.19]	1.00 [− 2.44;4.44]	0.34	853	4
GNP	6.08 [3.61;8.56]	2.54 [0.09;5]	1.00 [− 0.07;2.07]	0.19	565	5
GNS	3.49 [2.7;4.27]	1.00 [0.27;1.73]	1.00 [− 0.14;2.14]	0.09	879	5
GPS	5.35 [2.39;8.31]	4.48 [− 1.36;10.32]	1.00 [0.02;1.98]	0.15	775	20
NPS	12.52 [− 5.43;30.48]	15.00 [− 13.15;43.15]	1.69 [− 0.25;3.63]	0.38	863	4
GNPS	7.53 [1.85;13.21]	11.88 [2.15;21.61]	2.43 [− 0.13;5]	0.30	587	8
*A.oryzae*						
P	2.10 [− 3.12;7.31]	15.00 [− 30.57;60.57]	1.84 [− 1.95;5.62]	0.12	486	17
N	1.98 [− 2.97;6.93]	15.00 [− 39.09;69.09]	1.50 [− 1.37;4.38]	0.09	404	9
S	8.08 [6.8;9.37]	11.89 [10.26;13.51]	3.68 [2.44;4.92]	0.12	348	14
GN	27.79 [− 6.04;61.61]	15.00 [− 5.2;35.2]	2.07 [− 0.16;4.31]	0.87	370	24
GP	18.26 [14.83;21.69]	13.94 [12.2;15.69]	4.52 [2.75;6.29]	0.27	409	11
GS	20.8 [8.26;33.33]	15.00 [2.51;27.49]	1.58 [0.84;2.33]	0.25	405	40
NP	5.32 [2.71;7.94]	14.94 [11.16;18.73]	6.81 [− 2.68;16.29]	0.28	447	12
NS	4.35 [− 0.16;8.86]	15.00 [4.44;25.56]	3.96 [− 2.4;10.32]	0.24	219	7
PS	2.86 [1.67;4.04]	14.95 [11.69;18.22]	6.44 [− 0.72;13.61]	0.12	547	13
GNP	42.44 [30.93;53.94]	13.30 [10.49;16.11]	3.71 [1.9;5.52]	0.81	461	25
GNS	33.51 [− 38.6;105.61]	15.00 [− 27.87;57.87]	1.66 [− 1.18;4.5]	1.50	392	31
GPS	28.27 [12.71;43.84]	14.10 [7.23;20.96]	2.89 [0.89;4.89]	0.68	470	21
NPS	15.75 [7.81;23.7]	14.87 [11.00;18.75]	6.93 [− 3.32;17.18]	0.88	464	9
GNPS	54.86 [50.16;59.56]	8.27 [7.5;9.05]	5.03 [3.02;7.05]	1.09	513	119
*A.clavatus*						
N	2.25 [− 0.2;4.7]	1.13 [− 2.47;4.74]	1.00 [− 4.03;6.03]	0.28	489	11
P	3.63 [− 4.05;11.31]	7.76 [− 29.65;45.17]	1.00 [− 1.5;3.5]	0.23	613	12
S	6.17 [5.24;7.11]	15.00 [13.9;16.1]	8.63 [3.93;13.33]	0.13	406	13
GN	21.95 [− 14.49;58.39]	10.51 [− 27.85;48.88]	1.00 [− 0.55;2.55]	0.77	515	59
GP	8.88 [5.81;11.95]	6.89 [3.54;10.24]	2.16 [0.31;4.01]	0.34	696	12
GS	15.84 [− 19.66;51.34]	15.00 [− 54.84;84.84]	1.00 [− 0.57;2.57]	0.49	477	9
NP	4.90 [3.56;6.25]	15.00 [13.00;17.00]	8.55 [0.19;16.92]	0.18	417	9
NS	3.79 [2.82;4.75]	1.00 [0.18;1.82]	1.00 [− 0.29;2.29]	0.11	603	3
PS	3.51 [0.14;6.87]	15.00 [− 0.61;30.61]	2.12 [0.29;3.95]	0.09	658	11
GNP	48.28 [44.1;52.47]	6.08 [5.43;6.73]	5.02 [2.44;7.6]	1.23	362	14
GNS	10.09 [− 10.52;30.69]	15.00 [− 34.36;64.36]	1.33 [− 0.67;3.32]	0.35	668	6
GPS	14.15 [− 5.42;33.72]	15.00 [− 9.72;39.72]	1.89 [− 0.31;4.09]	0.46	646	7
NPS	19.50 [14.09;24.91]	15.00 [12.96;17.04]	8.22 [0.39;16.04]	0.71	617	25
GNPS	91.02 [88.39;93.65]	5.62 [5.43;5.8]	7.17 [5.65;8.69]	0.88	526	13
*A.nidulans*						
N	12.38 [− 18.74;43.5]	15.00 [− 41.73;71.73]	1.43 [− 1.28;4.15]	0.56	338	10
P	21.57 [14.2;28.94]	15.00 [12.51;17.49]	8.63 [− 1.99;19.24]	1.00	348	12
S	15.19 [11.1;19.28]	15.00 [12.9;17.1]	6.53 [1.8;11.26]	0.40	230	27
GN	80.75 [69.47;92.04]	9.69 [8.28;11.1]	3.42 [2.17;4.67]	1.41	515	105
GP	76.74 [74.05;79.43]	11.94 [11.64;12.25]	7.25 [6.09;8.41]	0.51	481	36
GS	22.66 [12.02;33.3]	14.48 [6.99;21.97]	2.12 [1.18;3.06]	0.31	548	86
NP	16.44 [6.74;26.13]	14.98 [10.63;19.33]	8.08 [− 8.03;24.18]	1.23	206	27
NS	9.61 [− 39.77;58.99]	15.00 [− 142.82;172.82]	1.02 [− 2.65;4.68]	0.68	422	10
PS	21.45 [14.1;28.8]	15.00 [12.03;17.97]	5.15 [1.5;8.8]	0.54	638	29
GNP	80.11 [76.24;83.97]	9.00 [8.56;9.45]	5.88 [4.51;7.24]	0.92	414	52
GNS	59.44 [47.05;71.83]	13.92 [11.23;16.62]	2.73 [2.04;3.42]	0.52	505	34
GPS	74.64 [72.24;77.05]	11.46 [11.16;11.76]	5.20 [4.63;5.76]	0.37	442	35
NPS	33.37 [24.81;41.94]	15.00 [13.09;16.91]	7.68 [1.37;13.98]	1.04	649	34
GNPS	85.46 [80.61;90.32]	9.67 [9.14;10.2]	6.28 [4.57;7.98]	1.10	328	27

Confidence intervals are indicated between brackets, N represents the number of objects at t = 1 h, while M represents the number of objects that could no longer be monitored between 2 and 16 h because the hypha had become too long or the object was obscured by hyphae of other objects. RMSE represents the root mean square error of the modelled data

The τ (time when *P* = 0.5 *P*_max_) of swelling of conidia and germ tube formation of the five tested aspergilli ranged between 4.24 and 11.59 h and 5.62 and 11.88 h, respectively, in GNPS (Tables 2, 3). *A. clavatus* showed the fastest germination, while *A. terreus* was the slowest. Heterogeneity in the swelling response in GNPS was lowest in the case of *A. clavatus* with a d of 6.40 after 16 h of incubation (i.e. t = 15), while *A. terreus* showed highest heterogeneity with a d of 2.18. Heterogeneity of germination showed a similar effect with *A. terreus* having the highest heterogeneity (d = 2.43) and *A. clavatus* having the lowest heterogeneity (d = 7.17) (Suppl. Figure. 2). Together, results show that presence of N, P, and S in combination with glucose result in hardly any germination (*A. terreus*) to almost full germination (*A. clavatus* and *A. nidulans*). The rate of germ tube formation also shows inter-species heterogeneity.

### Effect of amino acids on germination

**Fig. 1:**
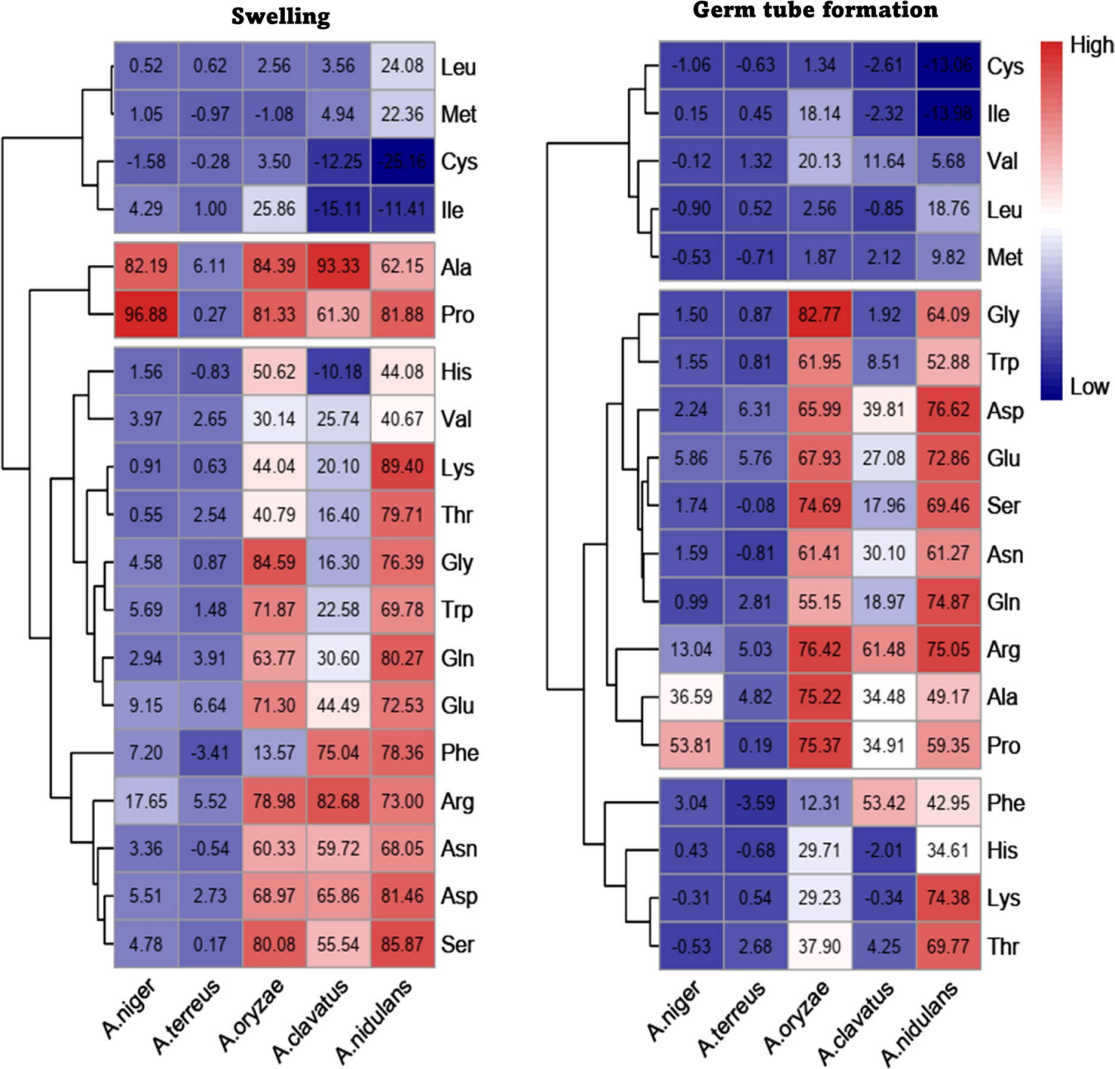
Heat map of normalized *P*_max_ of swelling and germ tube formation in response to amino acids taken up in PS (NaPO4 buffer, pH 6, MgSO4). To this end, the *P*_max_ of the medium containing the amino acid was subtracted from the *P*_max_ of the PS medium. Tyrosine was excluded from the data set because of the resulting low pH of the medium. Hierarchical clustering shows that low, intermediate and high swelling and germ tube inducing amino acids can be distinguished. Moreover, it is shown that clustering of the aspergilli based on swelling and germ tube formation does not follow phylogeny

OCelloScope imaging and data modelling was used to describe swelling and germ tube formation of conidia of 7 day-old cultures of *A. clavatus*, *A. nidulans*, *A. oryzae, A. terreus*, and *A. niger*. To this end, conidia of the 5 aspergilli were incubated in PS containing 10 mM of one of the 20 proteogenic amino acids. Alanine and proline highly induced swelling and germ tube formation except for *A. terreus* (Fig. 1, Suppl. Table 1, 2). *P*_max_ of swelling and germ tube formation of these amino acids was ≤ 6.11% and ≤ 4.28% in the case of *A. terreus*, while it ranged between 61.30 and 93.33% and between 34.48 and 75.37%, respectively, for the other aspergilli (Fig. 1). Leucine, methionine, cysteine, and isoleucine were the most lowly inducing amino acids in all aspergilli with a *P*_max_ ≤ 25.86. These amino acids, as well as valine, were also the most lowly inducing amino acids for germ tube formation with a *P*_max_ ≤ 20.13%.

Next, swelling and germ tube formation in GPS, NPS and GNPS was compared to that of PS combined with one of the amino acids (Tables 2, 3; Suppl. Tables 1, 2; Suppl. Figure 3). GPS (no nitrogen available) clustered with amino acids (both a carbon and nitrogen source) with intermediate inducing activity for swelling and germ tube formation, while NPS (no carbon source available) grouped with the low inducing amino acids (Suppl. Figure 3). GNPS (carbon and nitrogen source available) clustered with proline and alanine in the case of swelling and with arginine in the case of germ tube formation. The latter amino acid is also a high inducing amino acid. The swelling responses of *A. niger* and *A. terreus* clustered as well as those of *A. clavatus,* *A. oryzae* and *A.* *nidulans* (Suppl. Figure 2). In the case of germination, clustering was similar but *A.* *clavatus* now grouped with *A.* *niger* and *A.* *terreus.* Together, presence of glucose and nitrate is as effective in inducing germination in aspergilli as the highly inducing amino acids. Absence of either glucose or the nitrogen source reduces inducing capacity.

### Intra-species heterogeneity in swelling and germination of Aspergilli conidia

**Fig. 2:**
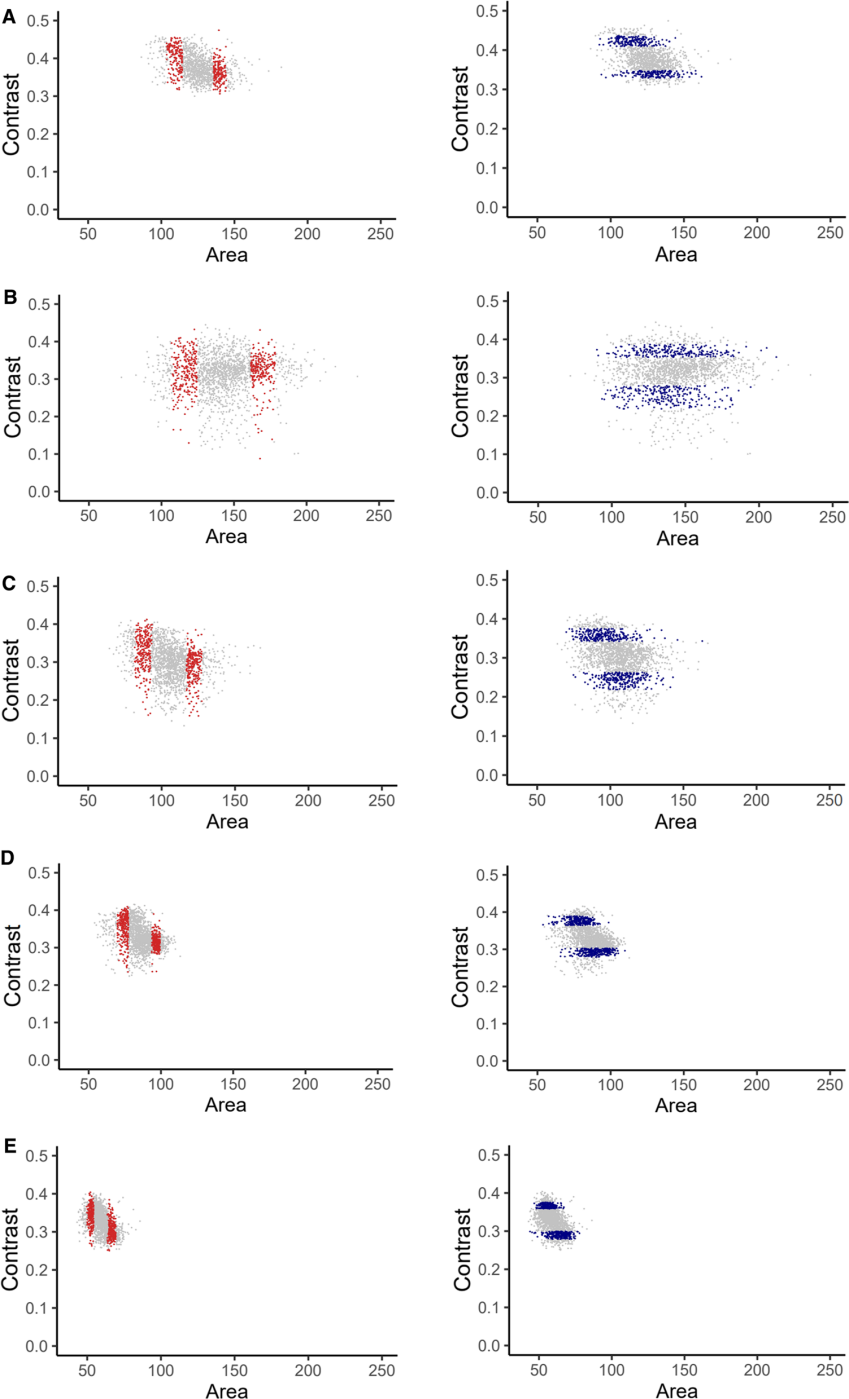
Scatter plots showing the 15% small and 15% large sized sub-populations (left) and 15% low and 15% high contrast sub-populations (right) of conida of representative replicates of *A.* *niger* (**A**), *A.* *oryzae* (**B**), *A.* *clavatus* (**C**), *A.* *nidulans* (**D**), and *A.* *terreus* (**E**)

Resting conidia of *Aspergillus* *niger* show highest variation in size, contrast and circular variance when compared to variation in circularity, compactness, granularity and moment gray (all object properties within the oCelloScope software). We here chose to analyse germination dynamics of sub-populations of spores differing in size and contrast. Spores differing in size have a different surface area to volume ratio, while spores with high contrast may have a denser cytoplasm and or cell wall. After removing the 5% smallest and largest conidia of each dataset (to remove outliers), the 15% smallest and 15% largest conidia were selected as well as the sub-populations consisting of the 15% conidia with the lowest and 15% with the highest contrast (Fig. 2). Statistical differences in swelling and germ tube formation dynamics were neither observed when the sub-populations of small and large conidia of *A.* *terreus* were compared nor when the subpopulations with high and low contrast were compared (Supplemental Table 3). In contrast, differences were observed in the subpopulations of conidia of the other aspergilli (Table 4; Supplemental Table 3). For instance, the sub-population of large and small conidia of *A. niger* showed a *P*_max_ of germ tube formation of 45.53 and 19.71%, respectively, when incubated in 10 mM proline (Table 4). In the case of alanine both the *P*_max_ of swelling and germ tube formation was different with 56.54 and 43.33% and 28.14 and 13.62% for the large and small populations, respectively. Together data show that sub-populations of conidia differing in size or contrast can behave differently within a spore population. If differences were observed, the large conidia showed a higher *P*_max_. No consistent trend was observed in the case of contrast.

**Table 4:** Parameter estimates of the asymmetrical model describing germination of sub-populations of small and large *A.* *niger* conidia and its conidia with low and high contrast

Variable	AA	Contrast	Size	*P*_max_ (%)	(h)	d (−)	RMSE	N	M
Swelling	Ala		Large	56.54 [49.63;63.45]A	5.4 [4.27;6.53]	4.03 [0.88;7.19]	2.08	68	19
Swelling	Ala		Small	43.33 [37.97;48.69]B	6.67 [5.53;7.81]	4.35 [1.34;7.36]	1.45	166	18
Swelling	Arg		Large	11.41 [− 3.86;26.68]	14.97 [4.4;25.54]	5.43 [− 8.45;19.3]	1.13	79	6
Swelling	Arg		Small	6.64 [− 0.6;13.87]	14.77 [7.07;22.47]	6.9 [− 12.31;26.11]	0.78	88	23
Swelling	Pro		Large	78.36 [76.51;80.22]	3.57 [3.37;3.78]	4.53 [3.49;5.58]	0.72	80	3
Swelling	Pro		Small	74.38 [69.98;78.78]	4.3 [3.77;4.84]	3.27 [2.02;4.53]	1.31	69	1
Swelling	Ala	High		55.88 [49.2;62.57]	7.04 [5.96;8.12]	2.58 [1.64;3.52]	0.91	144	9
Swelling	Ala	Low		64 [34.58;93.43]	7.86 [2.79;12.93]	1.86 [0.23;3.5]	2.06	65	9
Swelling	Arg	High		14.96 [− 82.17;112.09]	15 [− 185.58;215.58]	1 [− 3.63;5.63]	1.24	103	24
Swelling	Arg	Low		19.3 [− 11.11;49.71]	15 [− 3.04;33.04]	3.15 [− 2.59;8.9]	1.11	67	3
Swelling	Pro	High		74.97 [60.67;89.27]	4.99 [3.32;6.65]	1.6 [0.81;2.4]	1.39	52	2
Swelling	Pro	Low		77.7 [68.36;87.04]	3.93 [3.01;4.85]	1.78 [1.01;2.56]	1.38	115	5
Germ tube formation	Ala		Large	28.41 [20.57;36.24]A	15 [13.19;16.81]	8.48 [1.34;15.63]	1.02	68	19
Germ tube formation	Ala		Small	13.62 [6.84;20.4]B	15 [11.29;18.71]	6.07 [− 0.39;12.53]	0.58	166	18
Germ tube formation	Arg		Large	5.7 [2.65;8.75]	15 [11.27;18.73]	7.02 [− 2.36;16.4]	0.32	79	6
Germ tube formation	Arg		Small	4.01 [0.84;7.18]	14.89 [9.46;20.32]	7.4 [− 8.36;23.15]	0.36	88	23
Germ tube formation	Pro		Large	45.53 [29.68;61.38]A	14.42 [11.39;17.46]	4.55 [1.78;7.33]	1.06	80	3
Germ tube formation	Pro		Small	19.71 [13.22;26.19]B	15 [12.27;17.73]	4.98 [2.13;7.84]	0.43	69	1
Germ tube formation	Ala	High		20.98 [15.29;26.66]	15 [13.09;16.91]	6.87 [2.33;11.41]	0.57	144	9
Germ tube formation	Ala	Low		19.49 [12.85;26.14]	15 [12.49;17.51]	6.22 [1.57;10.86]	0.59	65	9
Germ tube formation	Arg	High		4.71 [− 8.49;17.91]	15 [− 19.36;49.36]	2.89 [− 5.9;11.68]	0.44	103	24
Germ tube formation	Arg	Low		4.01 [0.94;7.08]	15 [9.73;20.27]	7.27 [− 7.13;21.66]	0.33	67	3
Germ tube formation	Pro	High		24.15 [18.56;29.75]	14.39 [12.61;16.17]	5.74 [2.76;8.72]	0.51	52	2
Germ tube formation	Pro	Low		35.95 [28.96;42.95]	15 [13.27;16.73]	4.48 [3.11;5.85]	0.40	115	5

Conidia were incubated in 25 mM NaPO_4_ buffer pH 6.0, 2 mM MgSO_4_, and 10 mM alanin, arginine or proline. Confidence intervals are indicated beteen brackets, N represents the number of objects at t = 1 h, while M represents the number of objects that could no longer be monitored between 2 and 16 h because the hypha had become too long or the object was obscured by hyphae of other objects. RMSE represents the root mean square error of the modelled data

## Discussion

Aspergilli are abundant in nature and may therefore compete for substrates. Here we assessed the germination dynamics of spores of five aspergilli. Results show that these aspergilli differ in their germination response in water in the absence or presence of (in)organic nutrients. In addition, it is shown that subpopulations of spores of the aspergilli can have different germination responses to these environmental conditions. Data thus suggest that these fungi have evolved bet hedging mechanisms to maintain themselves in a dormant stress-resistant state on the one hand and to colonize a substrate on the other hand by a transition to a stress sensitive actively growing filamentous state.

Conidia leave their dormant stress-resistant state when they germinate. Previously, it was shown that conidia of *Cladosporium* *halotolerans* and *Penicillium* *rubens* germinate in pure water (Segers et al. 2017) and thus do not sense availability of nutrients before deciding to germinate. So far, this response seemed to be the exception since the majority of conidia of other fungi, including *A.* *niger* (Ijadpanahsaravi et al. 2021), *A.* *nidulans* (Osherov and May 2001) and *Penicillium* *roqueforti* (Punt et al. 2022) only germinate when (in)organic nutrients are available. Indeed, we here showed that the fast majority of the conidia of *A.* *terreus* and *A.* *oryzae* only germinate in the presence of organic nutrients. In contrast, 25% and 40% of the *A.* *clavatus* and *A.* *nidulans* spores had formed germ tubes, respectively, in the presence of only water. These data imply that *A.* *clavatus* and *A.* *nidulans* have a different germination strategy than the three other tested aspergilli. *A.* *clavatus* and *A.* *nidulans* simply start germinating when water is available, thus taking the risk of aborted growth when nutrients are absent in the environment. On the other hand, this strategy may result in fast colonization of the substrate when nutrients are present and thus would give a competitive advantage to conidia of other fungi that do sense the nutritional status before initiating germination. It should be noted that it came to a surprise that the *A.* *nidulans* conidia germinated in water since these spores were reported only to germinate in the presence of nutrients (Osherov and May 2001). Possibly, the genetic background, the pre-culture medium, the growth period, and the density of inoculation impact the capacity to germinate in water.

Particular (in)organic carbon and nitrogen sources increase the incidence of germination of *A.* *niger* conidia and/or support their outgrowth, while others do neither of both (Hayer et al. 2013, 2014; Ijadpanahsaravi et al. 2021). For instance, proline and alanine highly induce swelling and germ tube formation in *A.* *niger*, while cysteine, glutamine, histidine, leucine, lysine, methionine, threonine, tyrosine and valine are classified as low inducing amino acids (Ijadpanahsaravi et al. 2021). Alanine and proline also highly induced swelling and germ tube formation in the other aspergilli except for *A.* *terreus.* Clustering revealed that cysteine, leucine, methionine, and isoleucine are the least swelling-inducing amino acids of the aspergilli. These amino acids, as well as valine, were also the most lowly-germ-tube-inducing amino acids. Other amino acids showed different responses between aspergilli. *A.* *niger* and *A.* *terreus* clustered when swelling incidence on different amino acids was compared, while *A.* *clavatus,* *A.* *oryzae* and *A.* *nidulans* formed a second cluster. Clustering was similar when germ tube incidence was assessed but *A.* *clavatus* now grouped with *A.* *niger* and *A.* *terreus.* Notably, this clustering does not follow phylogeny (Houbraken et al. 2020) and suggests that germination responses on (in)organic nutrients have evolved relatively late in evolution. Data also strongly indicate that aspergilli have different competitive potential on different substrates depending on the absence or presence of (in)organic nutrients. Thus, competition not only takes place during vegetative growth but also during germination of conidia.

Conidia size of *A.* *niger,* *A.* *clavatus,* *A.* *nidulans* and *A.* *oryzae* was shown to impact germination. The large spores seem to be more responsive to inducers of germination than the small spores. The underlying mechanism is not yet known. Possibly, the sub-population of large conidia has a larger number of nutrient sensors because of the larger surface area of the plasma membrane. The relatively small size difference within the population of conidia of *A.* *terreus* may explain why a size effect on germination was not found in this *Aspergillus*. Conidia of *A.* *nidulans* and *A.* *oryzae* with high contrast responded differently to inducing amino acids when compared to spores with low contrast, a phenomenon not observed in the other aspergilli. Future studies should reveal the mechanisms underlying the different germination responses of sub-populations of conidia. These sub-populations can be selected by cell sorting, after which their molecular composition can be determined. This may lead to proteins or other components that are involved in the different germination dynamics of sub-populations of spores. Previously, it was shown that conidia of a single culture are heterogeneous in cell wall composition and in composition of RNA and proteins (Bleichrodt et al. 2013, 2020; Teertstra et al. 2017). For instance, G- or Ras-proteins (Fortwendel et al. 2004, 2008; Lafon et al. 2005) may be more abundant in more responsive sub-populations.

Together, it is tempting to speculate that *Aspergillus* species form conidia of different size or contrast to accommodate different germination responses within the spore population, thereby providing a bet hedging mechanism. The heterogeneity in spore germination responses adds to the heterogeneity of aspergilli in size and gene expression between micro-colonies (de Bekker et al. 2011), in expression between zones of micro-colonies (Tegelaar et al. 2020a), in secretion and stress resistance between hyphae within zones of micro-colonies (Tegelaar et al. 2020b), in composition of conidia (Bleichrodt et al. 2013, 2020; Teertstra et al. 2017; Wang et al., 2021) and between growth and branching capacity of compartments of individual hyphae (Tegelaar et al. 2017).

## Supplementary Information

Below is the link to the electronic supplementary material.

Supplementary file1 (DOCX 23940 KB)
