# Supplementary material for: Inter- and intra-species heterogeneity in germination of Aspergillus conidia
Source: Antonie Van Leeuwenhoek. 2022 Jul 20;115(9):1151–64. doi: 10.1007/s10482-022-01762-4 (PMC9363317; doi:10.1007/s10482-022-01762-4)
Supplement: Supplementary file 1 — Supplementary file1 (DOCX 23940 KB) [file 10482_2022_1762_MOESM1_ESM.docx]

**Supplemental Material**

**ssconidia**

Maryam Ijadpanahsaravi, Wieke R. Teertstra, Han A. B. Wösten^*^

Microbiology, Department of Biology, Utrecht University, Padualaan 8, 3584 CH Utrecht, The Netherlands.

**Supplemental Table 1.** Parameter estimates of the asymmetrical model describing swelling of conidia in 25 mM NaPO_4_ buffer pH 6.0 (P), 2 mM MgSO_4_ (S), and 10 mM L-amino acid (AA), the latter described by their three letter code (IUPAC). The pH was 6 throughout the experiment, except for tyrosine that had a pH between 1 and 2. Confidence intervals are indicated between brackets, N represents the number of objects at t = 1 h, while M represents the number of objects that could no longer be monitored between 2 and 16 h because the hypha had become too long or the object was obscured by hyphae of other objects. RMSE represents the root mean square error of the modelled data.

| **AA** | **Pmax (%)** | **Ꚍ (h)** | **d (-)** | **RMSE** | **N** | **M** |
| --- | --- | --- | --- | --- | --- | --- |
| ***A. niger*** | | | | | | |
| no | 2.11 [1.07;3.15] | 2.75 [-0.39;5.89] | 1.00 [-0.23;2.23] | 0.07 | 452 | 4 |
| Ala | 82.57 [79.42;85.73] | 5.45 [5.14;5.75] | 4.17 [3.27;5.08] | 0.90 | 567 | 83 |
| Arg | 19.39 [13.19;25.58] | 11.23 [7.91;14.55] | 3.06 [1.3;4.83] | 0.48 | 613 | 65 |
| Asn | 5.40 [-2.11;12.9] | 15.00 [-4.8;34.8] | 2.42 [-0.78;5.63] | 0.22 | 610 | 44 |
| Asp | 7.50 [-3.3;18.31] | 15.00 [-12.74;42.74] | 1.69 [-0.23;3.6] | 0.22 | 663 | 74 |
| Cys | 0.56 [0.24;0.88] | 2.06 [-0.97;5.1] | 1.40 [-2.06;4.87] | 0.05 | 735 | 4 |
| Gln | 4.99 [-2.7;12.69] | 15.00 [-26.47;56.47] | 1.16 [-0.11;2.43] | 0.11 | 684 | 24 |
| Glu | 11.07 [-11.67;33.8] | 15.00 [-46.32;76.32] | 1.05 [-0.47;2.56] | 0.32 | 612 | 109 |
| Gly | 6.59 [-0.8;13.98] | 15.00 [-4.34;34.34] | 1.92 [0.13;3.71] | 0.17 | 701 | 21 |
| His | 3.64 [-3.33;10.61] | 15.00 [-40.72;70.72] | 1.06 [-0.36;2.49] | 0.10 | 644 | 9 |
| Ile | 6.31 [-3.28;15.9] | 15.00 [-22.34;52.34] | 1.28 [-0.13;2.7] | 0.15 | 642 | 7 |
| Leu | 2.62 [-0.4;5.63] | 8.29 [-13.04;29.63] | 1.00 [-0.28;2.28] | 0.08 | 629 | 10 |
| Lys | 3.00 [-2.13;8.13] | 13.76 [-34.42;61.94] | 1.02 [-0.28;2.32] | 0.08 | 731 | 6 |
| Met | 3.14 [-7.23;13.51] | 15.00 [-84.25;114.25] | 1.03 [-1.34;3.4] | 0.14 | 649 | 5 |
| Phe | 9.16 [-6.64;24.97] | 7.17 [-20.87;35.22] | 1.00 [-1.15;3.15] | 0.48 | 630 | 79 |
| Pro | 96.95 [95.89;98.02] | 3.18 [3.1;3.26] | 4.16 [3.78;4.53] | 0.39 | 729 | 23 |
| Ser | 6.79 [4.8;8.78] | 7.59 [5.18;10.01] | 4.37 [-1.27;10.01] | 0.44 | 680 | 26 |
| Thr | 2.65 [-1.43;6.74] | 15.00 [-32.44;62.44] | 1.00 [-0.07;2.07] | 0.05 | 740 | 9 |
| Trp | 7.68 [3.52;11.84] | 13.16 [5.25;21.07] | 2.12 [0.91;3.34] | 0.14 | 762 | 9 |
| Tyr | 2.56 [1.77;3.35] | 7.57 [4.81;10.34] | 2.60 [0.51;4.69] | 0.09 | 507 | 9 |
| Val | 6.00 [-9.11;21.1] | 15.00 [-62.68;92.68] | 1.00 [-0.75;2.75] | 0.20 | 554 | 9 |
| ***A. terreus*** | | | | | | |
| no | 4.77 [4.1;5.45] | 1.00 [0.54;1.46] | 1.00 [0.28;1.72] | 0.08 | 872 | 7 |
| Ala | 10.59 [8.56;12.61] | 15.00 [13.59;16.41] | 8.11 [2.85;13.36] | 0.26 | 731 | 38 |
| Arg | 10.03 [-3.96;24.03] | 7.35 [-16.13;30.83] | 1.00 [-0.72;2.72] | 0.44 | 611 | 34 |
| Asn | 4.26 [2.88;5.64] | 1.00 [-0.05;2.05] | 1.00 [-0.64;2.64] | 0.16 | 586 | 4 |
| Asp | 7.37 [1.53;13.21] | 2.95 [-2.59;8.49] | 1.00 [-0.88;2.88] | 0.41 | 542 | 11 |
| Cys | 4.50 [3.81;5.2] | 1.00 [0.43;1.57] | 3.84 [-9.33;17.01] | 0.30 | 677 | 4 |
| Gln | 8.49 [4.12;12.85] | 1.00 [-0.66;2.66] | 1.00 [-1.61;3.61] | 0.52 | 700 | 13 |
| Glu | 11.09 [8.2;13.98] | 1.56 [0.54;2.57] | 1.00 [0.04;1.96] | 0.29 | 727 | 33 |
| Gly | 5.60 [3.16;8.05] | 1.00 [-0.41;2.41] | 1.00 [-1.21;3.21] | 0.29 | 730 | 13 |
| His | 3.98 [3.44;4.52] | 1.00 [0.44;1.56] | 3.40 [-5.28;12.08] | 0.23 | 502 | 4 |
| Ile | 5.72 [4.02;7.42] | 1.00 [0.04;1.96] | 1.00 [-0.5;2.5] | 0.20 | 652 | 5 |
| Leu | 5.36 [4.44;6.28] | 1.00 [0.12;1.88] | 2.17 [-2.21;6.55] | 0.32 | 684 | 3 |
| Lys | 5.37 [3.9;6.84] | 1.00 [0.12;1.88] | 1.00 [-0.39;2.39] | 0.18 | 820 | 3 |
| Met | 3.85 [1.91;5.8] | 1.00 [-0.62;2.62] | 1.00 [-1.55;3.55] | 0.23 | 605 | 8 |
| Phe | 1.52 [-0.11;3.15] | 1.71 [-2.94;6.35] | 1.12 [-3.47;5.7] | 0.19 | 383 | 4 |
| Pro | 5.03 [3.29;6.77] | 1.00 [-0.12;2.12] | 1.00 [-0.75;2.75] | 0.21 | 688 | 21 |
| Ser | 4.93 [4.02;5.85] | 1.05 [0.45;1.65] | 1.00 [0.1;1.9] | 0.11 | 830 | 8 |
| Thr | 7.19 [6.25;8.14] | 1.00 [0.34;1.66] | 2.42 [-1.71;6.55] | 0.35 | 676 | 2 |
| Trp | 6.18 [4.08;8.29] | 1.00 [-0.1;2.1] | 1.00 [-0.72;2.72] | 0.25 | 706 | 10 |
| Tyr | 4.26 [3.75;4.77] | 1.00 [0.4;1.6] | 2.41 [-1.37;6.2] | 0.19 | 589 | 4 |
| Val | 7.29 [2.49;12.09] | 1.00 [-1.13;3.13] | 1.00 [-2.34;4.34] | 0.57 | 560 | 4 |
| ***A. oryzae*** | | | | | | |
| no | 6.17 [1.11;11.23] | 15.00 [1.07;28.93] | 2.01 [0.57;3.45] | 0.13 | 786 | 10 |
| Ala | 85.35 [80.53;90.16] | 10.29 [9.75;10.83] | 4.89 [3.91;5.86] | 0.83 | 546 | 75 |
| Arg | 80.28 [78.76;81.8] | 7.03 [6.89;7.16] | 7.12 [6.19;8.06] | 0.47 | 698 | 134 |
| Asn | 62.78 [59.67;65.88] | 9.09 [8.62;9.56] | 4.61 [3.73;5.49] | 0.61 | 766 | 230 |
| Asp | 70.88 [68.27;73.49] | 6.79 [6.51;7.06] | 6.00 [4.61;7.4] | 0.78 | 649 | 148 |
| Cys | 9.45 [5.2;13.7] | 14.98 [11.39;18.57] | 6.14 [-0.83;13.11] | 0.40 | 893 | 13 |
| Gln | 66.01 [62.96;69.06] | 7.40 [7.04;7.77] | 6.17 [4.41;7.93] | 0.87 | 794 | 254 |
| Glu | 73.07 [70.54;75.59] | 5.43 [5.2;5.66] | 6.58 [4.95;8.21] | 0.85 | 752 | 195 |
| Gly | 85.54 [81.5;89.57] | 10.17 [9.72;10.62] | 5.16 [4.24;6.09] | 0.75 | 554 | 88 |
| His | 53.67 [45.77;61.56] | 15.00 [13.9;16.1] | 7.66 [4.06;11.26] | 0.97 | 820 | 90 |
| Ile | 30.43 [22.97;37.89] | 14.89 [13.07;16.72] | 7.82 [1.47;14.18] | 0.95 | 691 | 80 |
| Leu | 8.57 [4.56;12.57] | 15.00 [11.3;18.69] | 6.31 [-1.35;13.97] | 0.39 | 821 | 18 |
| Lys | 47.49 [41.74;53.24] | 15.00 [14.09;15.91] | 7.54 [4.67;10.41] | 0.69 | 770 | 127 |
| Met | 5.16 [2.75;7.58] | 14.91 [11.36;18.46] | 7.26 [-3.14;17.67] | 0.28 | 780 | 26 |
| Phe | 18.90 [13.23;24.56] | 15.00 [12.74;17.26] | 7.39 [0.58;14.2] | 0.66 | 813 | 76 |
| Pro | 82.48 [79.1;85.85] | 7.48 [7.12;7.83] | 4.39 [3.56;5.23] | 0.78 | 738 | 116 |
| Ser | 81.31 [75.48;87.13] | 10.64 [9.96;11.32] | 5.16 [3.82;6.5] | 1.02 | 710 | 134 |
| Thr | 44.44 [40.45;48.43] | 15.00 [14.34;15.66] | 8.43 [5.77;11.1] | 0.54 | 777 | 162 |
| Trp | 73.61 [67.19;80.04] | 15.00 [14.35;15.65] | 7.81 [5.59;10.03] | 0.80 | 841 | 112 |
| Tyr | 12.07 [7.31;16.84] | 15.00 [11.91;18.09] | 6.47 [-0.34;13.29] | 0.48 | 827 | 35 |
| Val | 34.45 [26.36;42.55] | 15.00 [13.22;16.78] | 7.23 [2.13;12.32] | 0.93 | 831 | 81 |
| ***A. clavatus*** | | | | | | |
| no | 14.93 [12.56;17.31] | 14.66 [13.22;16.11] | 4.73 [3.24;6.21] | 0.17 | 877 | 14 |
| Ala | 94.33 [90.18;98.48] | 12.22 [11.83;12.61] | 5.48 [4.69;6.27] | 0.59 | 857 | 31 |
| Arg | 85.27 [83.9;86.65] | 7.67 [7.53;7.81] | 5.09 [4.67;5.52] | 0.35 | 757 | 95 |
| Asn | 65.73 [60.52;70.93] | 15.00 [14.37;15.63] | 6.08 [4.88;7.28] | 0.48 | 878 | 82 |
| Asp | 70.96 [68.1;73.81] | 7.77 [7.42;8.12] | 5.01 [4;6.02] | 0.70 | 853 | 194 |
| Cys | 4.51 [-3.98;13.00] | 15.00 [-25.34;55.34] | 1.52 [-0.68;3.73] | 0.16 | 936 | 59 |
| Gln | 40.96 [38.1;43.81] | 12.94 [12.3;13.58] | 4.67 [3.86;5.47] | 0.29 | 849 | 300 |
| Glu | 52.78 [49.43;56.13] | 10.30 [9.66;10.93] | 3.82 [3.16;4.48] | 0.44 | 908 | 371 |
| Gly | 28.8 [18.39;39.22] | 14.77 [12.03;17.5] | 7.56 [-1.36;16.48] | 1.31 | 662 | 11 |
| His | 6.27 [2.41;10.12] | 15.00 [5.09;24.91] | 2.15 [0.95;3.35] | 0.10 | 972 | 14 |
| Ile | 2.08 [-1.94;6.11] | 15.00 [-20.65;50.65] | 1.82 [-1.09;4.73] | 0.09 | 862 | 16 |
| Leu | 17.96 [11.84;24.08] | 14.77 [12.1;17.44] | 6.62 [0.23;13.01] | 0.66 | 881 | 15 |
| Lys | 32.03 [28.43;35.63] | 11.98 [10.9;13.07] | 4.26 [3.06;5.45] | 0.40 | 978 | 12 |
| Met | 19.13 [12.92;25.34] | 14.81 [12.28;17.33] | 6.71 [0.48;12.94] | 0.68 | 860 | 16 |
| Phe | 78.77 [69.35;88.19] | 15.00 [14.02;15.98] | 5.83 [4.17;7.49] | 0.82 | 744 | 63 |
| Pro | 67.08 [63.41;70.76] | 15.00 [14.58;15.42] | 6.8 [5.75;7.85] | 0.39 | 919 | 127 |
| Ser | 62.18 [54.82;69.55] | 15.00 [14.12;15.88] | 7.82 [4.8;10.84] | 0.92 | 950 | 30 |
| Thr | 28.88 [19.48;38.28] | 14.71 [12.19;17.24] | 6.85 [0.24;13.46] | 1.07 | 898 | 15 |
| Trp | 34.14 [29.49;38.79] | 15.00 [13.97;16.03] | 7.27 [4.28;10.27] | 0.54 | 903 | 17 |
| Tyr | 1.00 [0.31;1.69] | 5.09 [-3.18;13.36] | 1.00 [-0.12;2.12] | 0.03 | 931 | 11 |
| Val | 36.83 [28.85;44.8] | 14.97 [13.36;16.59] | 7.76 [2.3;13.21] | 0.99 | 897 | 21 |
| ***A. nidulans*** | | | | | | |
| no | 44.6 [23.87;65.33] | 15.00 [10.49;19.51] | 4.26 [0.94;7.59] | 1.21 | 758 | 29 |
| Ala | 79.03 [73.33;84.73] | 15.00 [14.48;15.52] | 9.12 [6.62;11.61] | 0.83 | 770 | 59 |
| Arg | 85.04 [81.54;88.54] | 9.01 [8.63;9.39] | 6.01 [4.8;7.22] | 0.85 | 602 | 79 |
| Asn | 82.3 [71.34;93.26] | 13.87 [12.71;15.03] | 5.18 [3.48;6.88] | 1.05 | 735 | 122 |
| Asp | 89.73 [85.08;94.38] | 8.34 [7.86;8.82] | 4.23 [3.36;5.09] | 0.93 | 548 | 68 |
| Cys | 30.66 [7.23;54.09] | 15.00 [6.44;23.56] | 3.46 [-0.1;7.02] | 1.07 | 929 | 72 |
| Gln | 89.07 [85.85;92.29] | 10.31 [9.97;10.65] | 5.82 [4.93;6.71] | 0.65 | 676 | 88 |
| Glu | 84.78 [81.43;88.13] | 9.10 [8.72;9.47] | 4.75 [4.01;5.49] | 0.67 | 634 | 102 |
| Gly | 86.92 [75.44;98.4] | 15.00 [13.89;16.11] | 5.52 [3.89;7.14] | 0.93 | 721 | 93 |
| His | 69.02 [51.89;86.15] | 15.00 [12.27;17.73] | 3.54 [2.33;4.76] | 0.80 | 623 | 72 |
| Ile | 38.28 [19.87;56.69] | 15.00 [10.64;19.36] | 4.76 [0.42;9.1] | 1.24 | 815 | 51 |
| Leu | 57.94 [44.81;71.07] | 15.00 [13.24;16.76] | 6.64 [2.52;10.76] | 1.35 | 748 | 68 |
| Lys | 94.13 [83.22;105.05] | 13.88 [12.69;15.07] | 3.84 [3.07;4.62] | 0.72 | 725 | 38 |
| Met | 56.99 [35.69;78.3] | 15.00 [11.22;18.78] | 4.01 [1.66;6.37] | 1.16 | 809 | 52 |
| Phe | 88.01 [55.65;120.37] | 15.00 [11.09;18.91] | 3.73 [1.74;5.72] | 1.61 | 648 | 81 |
| Pro | 89.96 [80.65;99.26] | 13.29 [12.31;14.26] | 4.43 [3.41;5.45] | 0.83 | 729 | 87 |
| Ser | 92.17 [79.85;104.5] | 15.00 [13.83;16.17] | 5.1 [3.7;6.5] | 0.91 | 738 | 77 |
| Thr | 88.76 [79.37;98.16] | 14.44 [13.54;15.33] | 5.44 [4.06;6.83] | 0.85 | 688 | 69 |
| Trp | 83.26 [72.46;94.06] | 15.00 [13.92;16.08] | 5.62 [3.96;7.28] | 0.90 | 629 | 62 |
| Tyr | 5.02 [1.29;8.76] | 1.00 [-1.4;3.4] | 1.00 [-2.77;4.77] | 0.45 | 816 | 62 |
| Val | 67.13 [46.34;87.92] | 15.00 [12.15;17.85] | 4.63 [1.99;7.27] | 1.35 | 693 | 40 |

**Supplemental Table 2**. Parameter estimates of the asymmetrical model describing germtube formation of conidia in 25 mM NaPO_4_ buffer pH 6.0 (P), 2 mM MgSO_4_ (S), and 10 mM L-amino acid (AA), the latter described by their three letter code (IUPAC). The pH was 6 throughout the experiment, except for tyrosine that had a pH between 1 and 2. Confidence intervals are indicated between brackets, N represents the number of objects at t = 1 h, while M represents the number of objects that could no longer be monitored between 2 and 16 h because the hypha had become too long or the object was obscured by hyphae of other objects. RMSE represents the root mean square error of the modelled data.

| AA | Pmax (%) | Ꚍ (h) | d (-) | RMSE | N | M |
| --- | --- | --- | --- | --- | --- | --- |
| ***A. niger*** | | | | | | |
| no | 1.20 [-0.43;2.83] | 15.00 [-6.77;36.77] | 2.08 [-0.34;4.5] | 0.04 | 452 | 4 |
| Ala | 37.35 [32.38;42.33] | 12.19 [11.11;13.27] | 5.41 [3.33;7.48] | 0.65 | 567 | 83 |
| Arg | 14.08 [10.08;18.08] | 14.28 [11.52;17.04] | 3.91 [2.18;5.63] | 0.24 | 613 | 65 |
| Asn | 2.77 [0.15;5.39] | 15.00 [5.41;24.59] | 3.77 [-1.1;8.64] | 0.13 | 610 | 44 |
| Asp | 3.41 [1.63;5.2] | 9.75 [4.71;14.8] | 3.14 [-0.58;6.86] | 0.19 | 663 | 74 |
| Cys | 0.15 [0.04;0.25] | 2.81 [-2.13;7.74] | 2.12 [-5.79;10.03] | 0.03 | 735 | 4 |
| Gln | 2.18 [-1.6;5.95] | 15.00 [-23.05;53.05] | 1.45 [-0.44;3.34] | 0.07 | 684 | 24 |
| Glu | 6.99 [-4.18;18.16] | 15.00 [-22.02;52.02] | 1.39 [-0.27;3.06] | 0.20 | 612 | 109 |
| Gly | 2.68 [1.24;4.13] | 15.00 [9.98;20.02] | 4.25 [0.74;7.76] | 0.08 | 701 | 21 |
| His | 1.62 [0.17;3.07] | 15.00 [4.46;25.54] | 3.08 [-0.05;6.2] | 0.06 | 644 | 9 |
| Ile | 1.35 [0.83;1.88] | 9.54 [6.07;13.02] | 4.04 [-0.7;8.78] | 0.08 | 642 | 7 |
| Leu | 0.31 [-1.65;2.28] | 15.00 [-177.3;207.3] | 1.00 [-3.34;5.34] | 0.03 | 629 | 10 |
| Lys | 0.89 [-0.69;2.47] | 15.00 [-21.63;51.63] | 1.55 [-0.54;3.64] | 0.03 | 731 | 6 |
| Met | 0.68 [0.44;0.92] | 14.99 [12.61;17.37] | 7.43 [0.53;14.33] | 0.03 | 649 | 5 |
| Phe | 4.20 [-5.8;14.19] | 8.23 [-35.61;52.07] | 1.00 [-1.67;3.67] | 0.27 | 630 | 79 |
| Pro | 54.36 [51.08;57.64] | 11.34 [10.87;11.81] | 7.12 [5.3;8.95] | 0.64 | 729 | 23 |
| Ser | 2.92 [1.85;3.99] | 9.71 [6.62;12.79] | 6.18 [-3.81;16.16] | 0.24 | 680 | 26 |
| Thr | 0.68 [0.04;1.32] | 15.00 [3.44;26.56] | 2.90 [-0.04;5.84] | 0.02 | 740 | 9 |
| Trp | 2.73 [0.5;4.95] | 15.00 [4.02;25.98] | 2.60 [0.49;4.71] | 0.07 | 762 | 9 |
| Tyr | 1.37 [-0.19;2.92] | 7.96 [-8.3;24.22] | 1.31 [-0.69;3.31] | 0.06 | 507 | 9 |
| Val | 1.08 [-0.02;2.18] | 1.21 [-2.23;4.66] | 1.00 [-3.58;5.58] | 0.11 | 554 | 9 |
| ***A. terreus*** | | | | | | |
| no | 4.41 [3.87;4.95] | 1.00 [0.61;1.39] | 1.00 [0.38;1.62] | 0.06 | 872 | 7 |
| Ala | 9.02 [6.89;11.15] | 15.00 [13.25;16.75] | 7.89 [1.75;14.03] | 0.27 | 731 | 38 |
| Arg | 9.22 [-6.61;25.05] | 9.61 [-27.09;46.32] | 1.00 [-0.72;2.72] | 0.37 | 611 | 34 |
| Asn | 3.64 [2.43;4.86] | 1.00 [-0.08;2.08] | 1.00 [-0.69;2.69] | 0.14 | 586 | 4 |
| Asp | 10.44 [-17.35;38.23] | 15.00 [-67.96;97.96] | 1.00 [-0.86;2.86] | 0.38 | 542 | 11 |
| Cys | 3.81 [3.27;4.35] | 1.00 [0.41;1.59] | 3.29 [-5.09;11.68] | 0.22 | 677 | 4 |
| Gln | 7.10 [4.21;9.98] | 1.00 [-0.31;2.31] | 1.00 [-1.06;3.06] | 0.35 | 700 | 13 |
| Glu | 9.92 [6.2;13.64] | 1.93 [0.18;3.68] | 1.00 [-0.19;2.19] | 0.34 | 727 | 33 |
| Gly | 5.24 [3.01;7.47] | 1.00 [-0.37;2.37] | 1.00 [-1.16;3.16] | 0.27 | 730 | 13 |
| His | 3.76 [3.09;4.44] | 1.00 [0.07;1.93] | 2.07 [-2.1;6.23] | 0.23 | 502 | 4 |
| Ile | 4.84 [3.8;5.88] | 1.00 [0.3;1.7] | 1.00 [-0.09;2.09] | 0.12 | 652 | 5 |
| Leu | 4.91 [3.9;5.92] | 1.00 [-0.07;2.07] | 1.92 [-2.19;6.02] | 0.32 | 684 | 3 |
| Lys | 4.93 [3.25;6.61] | 1.00 [-0.1;2.1] | 1.00 [-0.72;2.72] | 0.20 | 820 | 3 |
| Met | 3.73 [1.87;5.59] | 1.00 [-0.61;2.61] | 1.00 [-1.53;3.53] | 0.22 | 605 | 8 |
| Phe | 0.98 [-0.16;2.11] | 1.00 [-2.76;4.76] | 1.00 [-4.91;6.91] | 0.13 | 383 | 4 |
| Pro | 4.59 [3.13;6.05] | 1.21 [0.13;2.29] | 1.00 [-0.4;2.4] | 0.16 | 688 | 21 |
| Ser | 4.33 [3.47;5.18] | 1.00 [0.36;1.64] | 1.00 [0;2.00] | 0.10 | 830 | 8 |
| Thr | 6.97 [4.44;9.49] | 1.00 [-0.17;2.17] | 1.00 [-0.84;2.84] | 0.30 | 676 | 2 |
| Trp | 5.18 [3.58;6.79] | 1.00 [0.0;2.00] | 1.00 [-0.57;2.57] | 0.19 | 706 | 10 |
| Tyr | 3.99 [3.39;4.59] | 1.00 [0.23;1.77] | 2.23 [-1.8;6.25] | 0.21 | 589 | 4 |
| Val | 5.67 [2.84;8.5] | 1.00 [-0.61;2.61] | 1.00 [-1.53;3.53] | 0.34 | 560 | 4 |
| ***A. oryzae*** | | | | | | |
| no | 2.02 [-0.23;4.28] | 15.00 [0.71;29.29] | 2.89 [-0.76;6.53] | 0.08 | 786 | 10 |
| Ala | 75.72 [70.27;81.17] | 11.36 [10.7;12.02] | 5.34 [3.99;6.68] | 0.87 | 546 | 75 |
| Arg | 76.90 [75.68;78.11] | 7.20 [7.09;7.31] | 7.58 [6.72;8.44] | 0.38 | 698 | 134 |
| Asn | 62.19 [59.16;65.22] | 9.19 [8.72;9.65] | 4.72 [3.82;5.61] | 0.60 | 766 | 230 |
| Asp | 66.68 [64.55;68.8] | 6.93 [6.69;7.16] | 6.28 [4.99;7.58] | 0.64 | 649 | 148 |
| Cys | 3.33 [1.3;5.37] | 14.88 [9.99;19.77] | 6.10 [-3.38;15.59] | 0.19 | 893 | 13 |
| Gln | 56.06 [53.97;58.15] | 7.78 [7.48;8.08] | 6.81 [5.2;8.42] | 0.60 | 794 | 254 |
| Glu | 68.58 [66.22;70.94] | 5.73 [5.51;5.96] | 7.11 [5.34;8.88] | 0.79 | 752 | 195 |
| Gly | 83.12 [79.65;86.6] | 10.36 [9.97;10.76] | 5.54 [4.61;6.47] | 0.67 | 554 | 88 |
| His | 31.13 [24.79;37.46] | 15.00 [13.49;16.51] | 7.85 [2.62;13.08] | 0.80 | 820 | 90 |
| Ile | 19.79 [13.27;26.31] | 14.71 [12.2;17.22] | 7.33 [-0.36;15.02] | 0.80 | 691 | 80 |
| Leu | 4.53 [1.73;7.33] | 14.99 [9.87;20.11] | 5.62 [-2.32;13.56] | 0.23 | 821 | 18 |
| Lys | 30.66 [26.23;35.1] | 15.00 [13.89;16.11] | 7.00 [4.06;9.93] | 0.49 | 770 | 127 |
| Met | 3.85 [2.05;5.65] | 14.92 [11.36;18.47] | 7.23 [-3.07;17.52] | 0.21 | 780 | 26 |
| Phe | 14.08 [9.3;18.85] | 15.00 [12.43;17.56] | 7.23 [-0.14;14.61] | 0.55 | 813 | 76 |
| Pro | 75.87 [72.28;79.46] | 8.00 [7.57;8.43] | 4.22 [3.39;5.05] | 0.75 | 738 | 116 |
| Ser | 75.2 [70.95;79.46] | 11.12 [10.61;11.64] | 5.91 [4.56;7.25] | 0.78 | 710 | 134 |
| Thr | 39.15 [35.13;43.18] | 15.00 [14.25;15.75] | 8.83 [5.48;12.18] | 0.57 | 777 | 162 |
| Trp | 62.72 [58.41;67.04] | 15.00 [14.51;15.49] | 9.54 [6.94;12.14] | 0.66 | 841 | 112 |
| Tyr | 11.23 [6.71;15.74] | 15.00 [11.85;18.15] | 6.48 [-0.48;13.45] | 0.45 | 827 | 35 |
| Val | 21.74 [15.26;28.21] | 14.77 [12.47;17.06] | 6.97 [0.75;13.2] | 0.74 | 831 | 81 |
| ***A. clavatus*** | | | | | | |
| no | 3.15 [1.62;4.68] | 15.00 [10.77;19.23] | 5.14 [-0.03;10.3] | 0.11 | 877 | 14 |
| Ala | 36.54 [28.22;44.87] | 14.99 [13.35;16.63] | 9.20 [1.15;17.26] | 1.23 | 857 | 31 |
| Arg | 62.69 [61.51;63.87] | 10.43 [10.25;10.6] | 6.90 [6.25;7.54] | 0.27 | 757 | 95 |
| Asn | 32.3 [27.19;37.4] | 14.98 [13.84;16.13] | 8.76 [3.69;13.84] | 0.72 | 878 | 82 |
| Asp | 41.71 [39.27;44.15] | 10.69 [10.18;11.21] | 8.01 [5.36;10.66] | 0.58 | 853 | 194 |
| Cys | 0.62 [0.2;1.04] | 1.06 [-1.14;3.25] | 1.00 [-2.28;4.28] | 0.05 | 936 | 59 |
| Gln | 21.52 [20.4;22.64] | 15.00 [14.63;15.37] | 9.40 [7.49;11.31] | 0.17 | 849 | 300 |
| Glu | 29.38 [27.91;30.86] | 13.19 [12.78;13.61] | 6.29 [5.23;7.36] | 0.20 | 908 | 371 |
| Gly | 5.01 [1.33;8.69] | 14.99 [9.06;20.91] | 5.98 [-4.77;16.73] | 0.33 | 662 | 11 |
| His | 1.20 [-0.24;2.64] | 15.00 [-8.46;38.46] | 1.70 [0.06;3.33] | 0.03 | 972 | 14 |
| Ile | 0.90 [-2.33;4.13] | 15.00 [-96.95;126.95] | 1.00 [-1.52;3.52] | 0.04 | 862 | 16 |
| Leu | 2.33 [1.06;3.6] | 14.98 [10.66;19.31] | 6.28 [-2.6;15.17] | 0.12 | 881 | 15 |
| Lys | 2.82 [0.56;5.07] | 15.00 [5.19;24.81] | 3.06 [0.14;5.97] | 0.09 | 978 | 12 |
| Met | 5.20 [2.51;7.89] | 14.94 [10.77;19.12] | 6.00 [-1.68;13.68] | 0.25 | 860 | 16 |
| Phe | 54.89 [48.66;61.11] | 15.00 [14.19;15.81] | 9.54 [5.25;13.83] | 0.95 | 744 | 63 |
| Pro | 36.96 [33.75;40.16] | 15.00 [14.38;15.62] | 9.84 [6.36;13.33] | 0.50 | 919 | 127 |
| Ser | 20.54 [13.74;27.34] | 14.74 [12.24;17.24] | 7.60 [-0.69;15.89] | 0.87 | 950 | 30 |
| Thr | 7.27 [2.82;11.72] | 14.73 [9.69;19.77] | 5.76 [-2.86;14.38] | 0.41 | 898 | 15 |
| Trp | 11.39 [6.89;15.89] | 14.80 [11.73;17.88] | 6.70 [-0.86;14.26] | 0.49 | 903 | 17 |
| Tyr | 0.49 [0.19;0.79] | 1.32 [-0.84;3.48] | 1.00 [-1.53;3.53] | 0.03 | 931 | 11 |
| Val | 14.42 [8.43;20.41] | 14.76 [11.49;18.04] | 6.45 [-0.93;13.83] | 0.63 | 897 | 21 |
| ***A. nidulans*** | | | | | | |
| no | 30.33 [12.26;48.39] | 15.00 [9.18;20.82] | 4.22 [0.05;8.39] | 1.05 | 758 | 29 |
| Ala | 64.59 [57.2;71.97] | 15.00 [14.18;15.82] | 9.58 [5.22;13.94] | 1.13 | 770 | 59 |
| Arg | 82.62 [79.5;85.74] | 9.48 [9.13;9.83] | 6.41 [5.21;7.6] | 0.75 | 602 | 79 |
| Asn | 73.02 [63.88;82.16] | 14.78 [13.8;15.76] | 6.51 [4.25;8.77] | 0.96 | 735 | 122 |
| Asp | 83.71 [80.41;87] | 9.20 [8.83;9.57] | 5.23 [4.35;6.11] | 0.71 | 548 | 68 |
| Cys | 21.23 [1.67;40.79] | 15.00 [4.47;25.53] | 3.37 [-0.7;7.44] | 0.87 | 929 | 72 |
| Gln | 82.49 [79.91;85.07] | 11.44 [11.17;11.71] | 8.61 [7.1;10.12] | 0.59 | 676 | 88 |
| Glu | 81.09 [78.38;83.81] | 9.72 [9.4;10.03] | 5.57 [4.78;6.37] | 0.58 | 634 | 102 |
| Gly | 74.98 [67.86;82.1] | 15.00 [14.29;15.71] | 7.48 [5.27;9.69] | 0.85 | 721 | 93 |
| His | 54.44 [45.41;63.47] | 15.00 [13.58;16.42] | 5.25 [3.41;7.09] | 0.69 | 623 | 72 |
| Ile | 20.59 [10.06;31.13] | 15.00 [10.24;19.76] | 4.56 [0.35;8.76] | 0.67 | 815 | 51 |
| Leu | 43.40 [32.55;54.25] | 15.00 [13.08;16.92] | 6.87 [1.98;11.76] | 1.17 | 748 | 68 |
| Lys | 82.15 [75.63;88.66] | 15.00 [14.38;15.62] | 6.48 [5.11;7.86] | 0.65 | 725 | 38 |
| Met | 37.17 [22.91;51.42] | 15.00 [11.4;18.6] | 4.50 [1.43;7.56] | 0.89 | 809 | 52 |
| Phe | 60.25 [55;65.51] | 15.00 [14.35;15.65] | 7.44 [5.43;9.45] | 0.62 | 648 | 81 |
| Pro | 71.68 [65.64;77.72] | 15.00 [14.36;15.64] | 7.27 [5.42;9.13] | 0.70 | 729 | 87 |
| Ser | 78.72 [71.21;86.24] | 15.00 [14.27;15.73] | 6.99 [5.06;8.92] | 0.82 | 738 | 77 |
| Thr | 78.94 [69.66;88.21] | 15.00 [14.08;15.92] | 6.55 [4.47;8.62] | 0.94 | 688 | 69 |
| Trp | 67.17 [56.93;77.41] | 15.00 [13.83;16.17] | 6.97 [3.9;10.04] | 1.12 | 629 | 62 |
| Tyr | 4.34 [0.83;7.85] | 1.24 [-1.52;4.00] | 1.00 [-2.49;4.49] | 0.39 | 816 | 62 |
| Val | 34.29 [24.28;44.31] | 15.00 [12.49;17.51] | 5.25 [2.01;8.49] | 0.76 | 693 | 40 |

**Supplemental Table 3.** Parameter estimates of the asymmetrical model describing germination of sub-populations of small and large and conidia and conidia with low and high contrast of each of the five tested aspergilli. Conidia were incubated in 25 mM NaPO_4_ buffer pH 6.0, 2 mM MgSO_4_, and 10 mM alanine, arginine or proline. Confidence intervals are indicated between brackets, N represents the number of objects at t = 1 h, while M represents the number of objects that could no longer be monitored between 2 and 16 h because the hypha had become too long or the object was obscured by hyphae of other objects. RMSE represents the root mean square error of the modelled data.

| **Variable** | **AA** | **Contrast** | **Size** | **Pmax (%)** | **Ꚍ (h)** | **d (-)** | **RMSE** | **N** | **M** |
| --- | --- | --- | --- | --- | --- | --- | --- | --- | --- |
| ***A. oryzae*** | | | | | | | | | |
| Swelling | Ala |  | Large | 99.19[96.73;101.65] | 5.83[5.6;6.05]A | 5.33[4.34;6.32] | 0.83 | 87 | 1 |
| Swelling | Ala |  | Small | 95.23[91.85;98.6] | 8.73[8.42;9.04]B | 5.74[4.66;6.82] | 0.87 | 119 | 6 |
| Swelling | Arg |  | Large | 54.65[48.66;60.65] | 8.43[7.45;9.41]A | 3.88[2.26;5.5] | 1.10 | 100 | 11 |
| Swelling | Arg |  | Small | 68.84[55.64;82.03] | 12.59[10.85;14.33]B | 4.02[2.43;5.61] | 1.12 | 78 | 12 |
| Swelling | Pro |  | Large | 77.75[69.36;86.14] | 5.14[4.21;6.06] | 2.5[1.38;3.62] | 1.59 | 89 | 8 |
| Swelling | Pro |  | Small | 88.8[84.52;93.08] | 5.84[5.41;6.27] | 3.23[2.48;3.98] | 0.98 | 77 | 5 |
| Swelling | Ala | High |  | 85.6[78.99;92.2] | 9.42[8.75;10.09]A | 5.72[3.6;7.84] | 1.55 | 113 | 17 |
| Swelling | Ala | Low |  | 90.01[88.23;91.79] | 10.69[10.52;10.85]B | 6.87[6.22;7.52] | 0.41 | 115 | 10 |
| Swelling | Arg | High |  | 11.37[-4.25;26.98]A | 15[1.78;28.22] | 3.99[-3.73;11.7] | 0.76 | 60 | 19 |
| Swelling | Arg | Low |  | 82.46[78.6;86.32]B | 10.44[10.05;10.83] | 6.92[5.31;8.52] | 0.92 | 106 | 4 |
| Swelling | Pro | High |  | 63.78[55.04;72.52]A | 9.57[8.49;10.66]A | 9.81[0.15;19.48] | 2.72 | 68 | 11 |
| Swelling | Pro | Low |  | 89.81[85.11;94.51]B | 6.73[6.25;7.22]B | 4.34[3.09;5.6] | 1.26 | 64 | 5 |
| Germ tube formation | Ala |  | Large | 97.68[93.52;101.83] | 8.87[8.5;9.25]A | 5.61[4.39;6.83] | 1.03 | 87 | 1 |
| Germ tube formation | Ala |  | Small | 90.88[65.41;116.36] | 14.06[11.53;16.59]B | 4.26[2.18;6.34] | 1.69 | 119 | 6 |
| Germ tube formation | Arg |  | Large | 48.59[44.13;53.06] | 10.17[9.39;10.95]A | 5.56[3.41;7.72] | 0.91 | 100 | 11 |
| Germ tube formation | Arg |  | Small | 58.98[46.57;71.39] | 14.02[12.3;15.74]B | 5.08[2.84;7.33] | 1.06 | 78 | 12 |
| Germ tube formation | Pro |  | Large | 59.75[53.25;66.24] | 6.95[5.95;7.95] | 4.27[1.83;6.72] | 1.67 | 89 | 8 |
| Germ tube formation | Pro |  | Small | 70.06[60.59;79.53] | 7.68[6.47;8.9] | 3.49[1.69;5.3] | 1.74 | 77 | 5 |
| Germ tube formation | Ala | High |  | 84.01[75.35;92.66] | 9.84[8.96;10.73]A | 5.31[3;7.63] | 1.78 | 113 | 17 |
| Germ tube formation | Ala | Low |  | 90.23[88.69;91.76] | 11.15[11.01;11.29]B | 6.8[6.29;7.32] | 0.33 | 115 | 10 |
| Germ tube formation | Arg | High |  | 10.79[-5.03;26.6]A | 15[0.1;29.9] | 3.7[-3.45;10.84] | 0.70 | 60 | 19 |
| Germ tube formation | Arg | Low |  | 81.06[77.48;84.64]B | 10.64[10.27;11] | 6.89[5.43;8.35] | 0.83 | 106 | 4 |
| Germ tube formation | Pro | High |  | 33.56[-1.88;69]A | 12.09[-0.45;24.62] | 2.56[-1.32;6.43] | 1.78 | 68 | 11 |
| Germ tube formation | Pro | Low |  | 88[82.99;93.02]B | 6.91[6.39;7.44] | 4.4[3.04;5.77] | 1.33 | 64 | 5 |
| ***A. terreus*** | | | | | | | | | |
| Swelling | Ala |  | Large | 9.99[3.17;16.81] | 4.32[-1.11;9.75] | 1.79[-2.3;5.88] | 0.93 | 154 | 32 |
| Swelling | Ala |  | Small | 16.02[-26;58.04] | 15[-34.19;64.19] | 1.73[-2;5.46] | 0.84 | 173 | 41 |
| Swelling | Arg |  | Large | 8.04[-1.57;17.66] | 15[4.36;25.64] | 4.47[-3.9;12.84] | 0.55 | 80 | 13 |
| Swelling | Arg |  | Small | 7.27[-3.77;18.3] | 14.99[3.39;26.59] | 5.77[-11.99;23.52] | 0.88 | 191 | 46 |
| Swelling | Pro |  | Large | 8.75[-15.81;33.3] | 15[-46.3;76.3] | 1.46[-1.71;4.62] | 0.42 | 135 | 47 |
| Swelling | Pro |  | Small | 10.23[3.61;16.84] | 15[9.66;20.34] | 5.04[-0.71;10.79] | 0.44 | 97 | 11 |
| Swelling | Ala | High |  | 12.08[-27.28;51.43] | 15[-54.43;84.42] | 1.5[-2.31;5.31] | 0.69 | 179 | 40 |
| Swelling | Ala | Low |  | 12.96[-1.55;27.46] | 15[-3.84;33.84] | 1.96[0.06;3.87] | 0.32 | 136 | 62 |
| Swelling | Arg | High |  | 5.75[-22.37;33.87] | 15[-136.08;166.08] | 1[-2.49;4.49] | 0.36 | 188 | 68 |
| Swelling | Arg | Low |  | 4.76[-2.23;11.75] | 15[1.33;28.67] | 4.18[-4.83;13.19] | 0.36 | 74 | 20 |
| Swelling | Pro | High |  | 1.52[0.82;2.21] | 13.02[9.81;16.22] | 10.96[-15.17;37.09] | 0.16 | 72 | 4 |
| Swelling | Pro | Low |  | 5.47[-20.91;31.85] | 15[-133.95;163.95] | 1[-2.44;4.44] | 0.34 | 211 | 110 |
| Germ tube formation | Ala |  | Large | 8.76[-15.62;33.14] | 14.15[-67.75;96.04] | 1[-1.08;3.08] | 0.33 | 154 | 32 |
| Germ tube formation | Ala |  | Small | 10.87[-19.05;40.79] | 15[-40.02;70.02] | 1.61[-1.93;5.15] | 0.56 | 173 | 41 |
| Germ tube formation | Arg |  | Large | 7.67[-1.92;17.26] | 15[3.13;26.87] | 4.07[-3.22;11.35] | 0.48 | 80 | 13 |
| Germ tube formation | Arg |  | Small | 6.42[-1.96;14.8] | 14.83[5.48;24.17] | 6.65[-14.47;27.78] | 0.85 | 191 | 46 |
| Germ tube formation | Pro |  | Large | 7.73[-8.84;24.31] | 15[-27.78;57.78] | 1.61[-1.15;4.38] | 0.31 | 135 | 47 |
| Germ tube formation | Pro |  | Small | 5.05[-1.71;11.81] | 15[0.41;29.59] | 3.36[-2.12;8.85] | 0.27 | 97 | 11 |
| Germ tube formation | Ala | High |  | 8.03[-20.01;36.07] | 15[-67.62;97.62] | 1.33[-2.18;4.85] | 0.45 | 179 | 40 |
| Germ tube formation | Ala | Low |  | 8.39[3.87;12.9] | 15[9.71;20.29] | 3.87[1.02;6.72] | 0.21 | 136 | 62 |
| Germ tube formation | Arg | High |  | 1.87[-0.13;3.88] | 14.99[7.8;22.19]A | 7.83[-15.73;31.38] | 0.24 | 188 | 68 |
| Germ tube formation | Arg | Low |  | 1.12[0.72;1.52] | 1[-0.05;2.05]B | 6.17[-169.79;182.13] | 0.19 | 74 | 20 |
| Germ tube formation | Pro | High |  | 0.99[0.63;1.36] | 14[12.25;15.75] | 30[-77.76;137.76] | 0.10 | 72 | 4 |
| Germ tube formation | Pro | Low |  | 4.18[-16.93;25.29] | 15[-141.03;171.03] | 1[-2.6;4.6] | 0.27 | 211 | 110 |
| ***A. clavatus*** | | | | | | | | | |
| Swelling | Ala |  | Large | 102.73[93.38;112.08] | 9.47[8.59;10.36]A | 2.9[2.28;3.52] | 0.94 | 123 | 2 |
| Swelling | Ala |  | Small | 108.57[79.19;137.96] | 15[11.99;18.01]B | 3.27[2.21;4.32] | 1.12 | 81 | 1 |
| Swelling | Arg |  | Large | 97.08[84.67;109.5] | 12.86[11.65;14.07] | 3.77[2.88;4.67] | 0.91 | 101 | 11 |
| Swelling | Arg |  | Small | 81.1[47.69;114.51] | 15[10.49;19.51] | 3.35[1.67;5.02] | 1.31 | 142 | 11 |
| Swelling | Pro |  | Large | 77.74[70.65;84.84]A | 12.06[11.29;12.84]A | 4.63[3.53;5.73] | 0.82 | 93 | 5 |
| Swelling | Pro |  | Small | 31.24[23.41;39.07]B | 15[13.32;16.68]B | 7.93[2.27;13.59] | 0.94 | 48 | 13 |
| Swelling | Ala | High |  | 77.42[69.99;84.86] | 10.92[10.16;11.68]A | 8.96[4;13.92] | 1.96 | 67 | 0 |
| Swelling | Ala | Low |  | 97.22[77.39;117.06] | 15[13.04;16.96]B | 3.99[2.84;5.14] | 0.97 | 138 | 5 |
| Swelling | Arg | High |  | 91.37[38.23;144.5] | 15[7.76;22.24] | 2.83[1.07;4.59] | 1.71 | 156 | 11 |
| Swelling | Arg | Low |  | 38.44[23.5;53.37] | 15[12.23;17.77] | 6.67[0.54;12.8] | 1.43 | 92 | 23 |
| Swelling | Pro | High |  | 64.58[43.8;85.36] | 15[12.72;17.28] | 6.77[1.54;12.01] | 2.05 | 16 | 4 |
| Swelling | Pro | Low |  | 58.17[46.53;69.81] | 15[13.46;16.54] | 5.71[3.42;8.01] | 0.92 | 128 | 18 |
| Germ tube formation | Ala |  | Large | 26.29[5.33;47.24] | 15[7.26;22.74] | 3.94[-0.43;8.31] | 1.01 | 123 | 2 |
| Germ tube formation | Ala |  | Small | 16.4[4.9;27.9] | 15[9.27;20.73] | 5.11[-1.29;11.52] | 0.78 | 81 | 1 |
| Germ tube formation | Arg |  | Large | 14.25[7.93;20.58] | 15[11.61;18.39] | 5.78[0.57;10.99] | 0.51 | 101 | 11 |
| Germ tube formation | Arg |  | Small | 13.29[6.81;19.76] | 14.99[11.49;18.5] | 6.53[-0.82;13.88] | 0.61 | 142 | 11 |
| Germ tube formation | Pro |  | Large | 49.19[42;56.39]A | 15[14.05;15.95] | 8.69[4.72;12.67] | 0.96 | 93 | 5 |
| Germ tube formation | Pro |  | Small | 5.53[0.54;10.51]B | 14.82[8.55;21.1] | 7.16[-9.85;24.18] | 0.55 | 48 | 13 |
| Germ tube formation | Ala | High |  | 12.16[2.92;21.39] | 15[9.2;20.8] | 5.76[-3.1;14.62] | 0.74 | 67 | 0 |
| Germ tube formation | Ala | Low |  | 17.41[12.78;22.04] | 15[13.27;16.73] | 8.64[1.5;15.79] | 0.62 | 138 | 5 |
| Germ tube formation | Arg | High |  | 13.76[6.28;21.24] | 14.98[11.11;18.86] | 6.68[-1.94;15.3] | 0.73 | 156 | 11 |
| Germ tube formation | Arg | Low |  | 6.58[-1;14.17] | 14.85[6.55;23.15] | 6.55[-11.41;24.5] | 0.74 | 92 | 23 |
| Germ tube formation | Pro | High |  | 33.85[20.03;47.67] | 14.84[12.12;17.56] | 8.21[-1.95;18.38] | 1.80 | 16 | 4 |
| Germ tube formation | Pro | Low |  | 25.66[17.67;33.64] | 15[12.85;17.15] | 7.16[1.48;12.85] | 0.85 | 128 | 18 |
| ***A. nidulans*** | | | | | | | | | |
| Swelling | Ala |  | Large | 87.78[81.25;94.3]A | 11.49[10.92;12.06] | 9.08[5.42;12.74] | 1.63 | 64 | 9 |
| Swelling | Ala |  | Small | 73.02[63.8;82.23]B | 12.95[11.98;13.93] | 6.1[3.76;8.43] | 1.27 | 143 | 39 |
| Swelling | Arg |  | Large | 96.53[87.76;105.31] | 14.78[14.11;15.45] | 6.24[4.94;7.54] | 0.82 | 106 | 3 |
| Swelling | Arg |  | Small | 85.4[76.14;94.66] | 14.87[14.12;15.62] | 7.31[5.19;9.42] | 1.04 | 106 | 4 |
| Swelling | Pro |  | Large | 77.12[54.67;99.57] | 11.82[9.2;14.43] | 3.97[1.31;6.64] | 2.22 | 104 | 17 |
| Swelling | Pro |  | Small | 61.72[51.46;71.98] | 11.3[9.9;12.7] | 4.79[2.37;7.21] | 1.45 | 56 | 27 |
| Swelling | Ala | High |  | 77.91[62.72;93.11]A | 13.57[12.14;15.01] | 6.79[2.72;10.86] | 2.09 | 141 | 26 |
| Swelling | Ala | Low |  | 51.3[43.43;59.17]B | 12.46[11.28;13.63] | 7.11[2.93;11.29] | 1.41 | 43 | 13 |
| Swelling | Arg | High |  | 88.76[68.36;109.16] | 15[13.18;16.82] | 5.39[3.05;7.73] | 1.49 | 163 | 9 |
| Swelling | Arg | Low |  | 77.5[66.03;88.97] | 15[13.87;16.13] | 5.81[4.06;7.57] | 0.92 | 93 | 4 |
| Swelling | Pro | High |  | 36.51[33.19;39.84]A | 10.83[10.09;11.58] | 7.23[4.01;10.44] | 0.77 | 59 | 35 |
| Swelling | Pro | Low |  | 78.86[71.21;86.51]B | 10.86[10.03;11.7] | 4.53[3.18;5.89] | 1.10 | 110 | 18 |
| Germ tube formation | Ala |  | Large | 81.86[74.44;89.28]A | 14.59[14.01;15.18] | 10.41[6.64;14.17] | 1.29 | 64 | 9 |
| Germ tube formation | Ala |  | Small | 59.14[52.3;65.98]B | 14.84[14.05;15.64] | 7.36[5.06;9.65] | 0.78 | 143 | 39 |
| Germ tube formation | Arg |  | Large | 70.34[64.83;75.85] | 15[14.5;15.5] | 9.65[7.03;12.28] | 0.83 | 106 | 3 |
| Germ tube formation | Arg |  | Small | 58.31[48.59;68.04] | 15[13.92;16.08] | 8.91[4.14;13.67] | 1.34 | 106 | 4 |
| Germ tube formation | Pro |  | Large | 44.85[27.93;61.77] | 14.13[11.3;16.95] | 6.15[0.29;12.01] | 1.82 | 104 | 17 |
| Germ tube formation | Pro |  | Small | 29.88[26.29;33.48] | 12.99[12.2;13.78] | 14.04[3.45;24.62] | 0.93 | 56 | 27 |
| Germ tube formation | Ala | High |  | 68.55[58.91;78.19]A | 15[14.11;15.89] | 10.12[4.94;15.29] | 1.51 | 141 | 26 |
| Germ tube formation | Ala | Low |  | 49.31[42.05;56.56]B | 13.31[12.26;14.36] | 8.76[3.44;14.07] | 1.37 | 43 | 13 |
| Germ tube formation | Arg | High |  | 63.12[54.63;71.61] | 15[14.12;15.88] | 8.53[5.01;12.06] | 1.11 | 163 | 9 |
| Germ tube formation | Arg | Low |  | 64.69[54.34;75.04] | 15[13.92;16.08] | 7.73[4.31;11.14] | 1.20 | 93 | 4 |
| Germ tube formation | Pro | High |  | 13.88[-3.21;30.97] | 12.16[-0.49;24.82] | 3.18[-3.61;9.96] | 1.13 | 59 | 35 |
| Germ tube formation | Pro | Low |  | 51.57[28.12;75.03] | 14.81[11.4;18.22] | 6[0.04;11.96] | 2.07 | 110 | 18 |


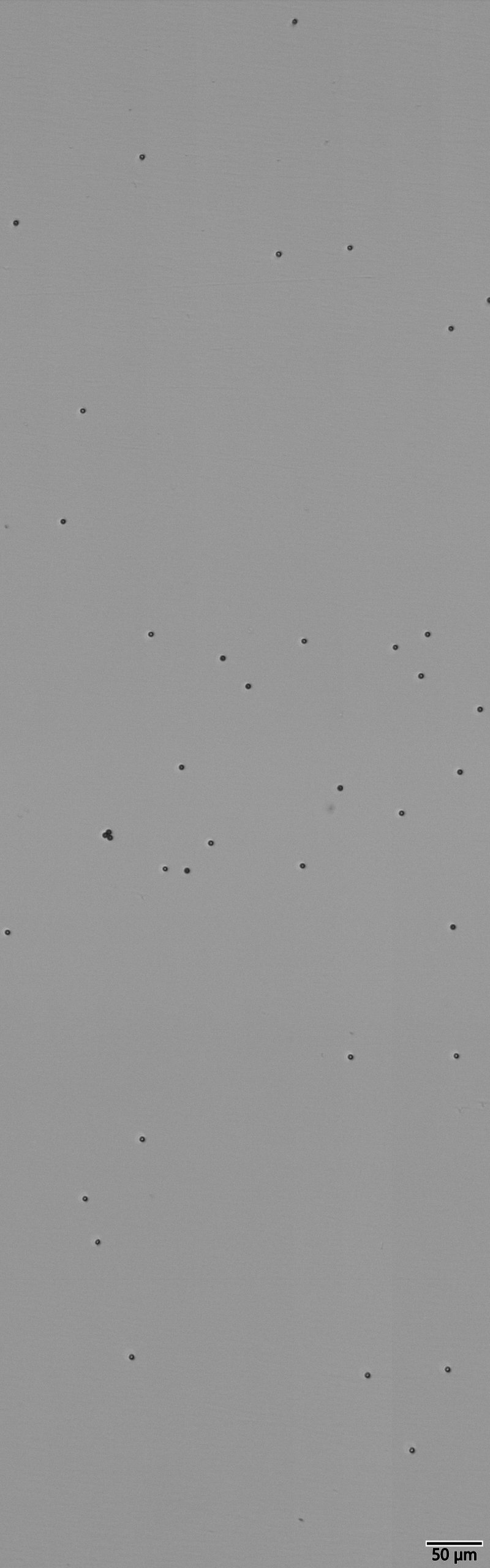


**A**


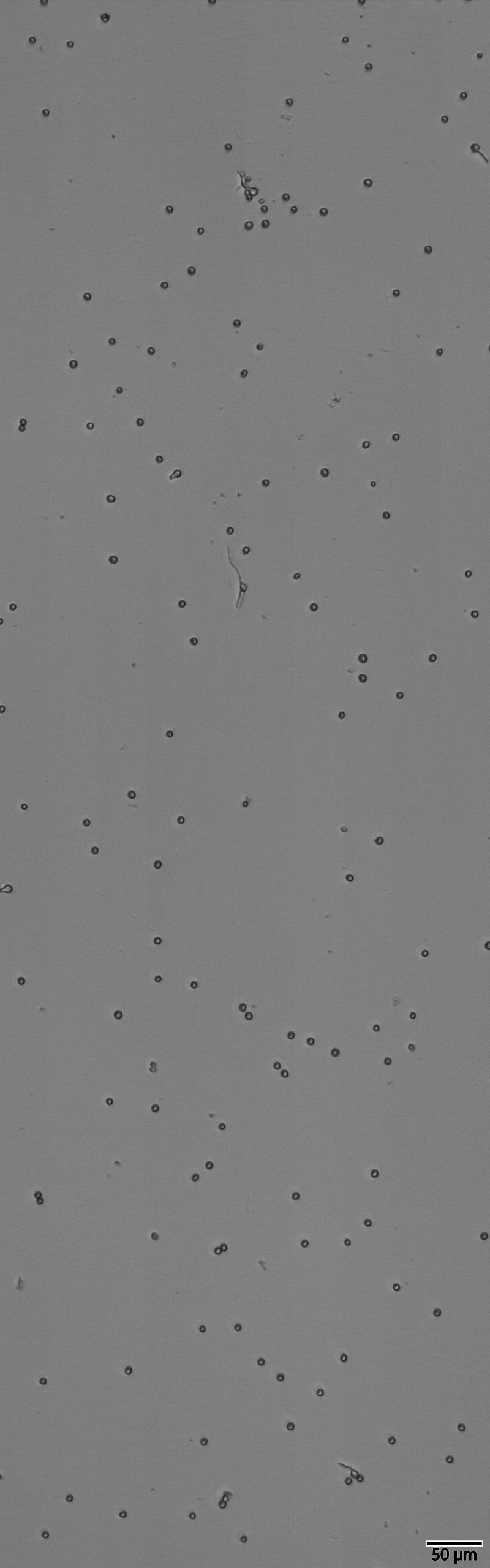


**C**


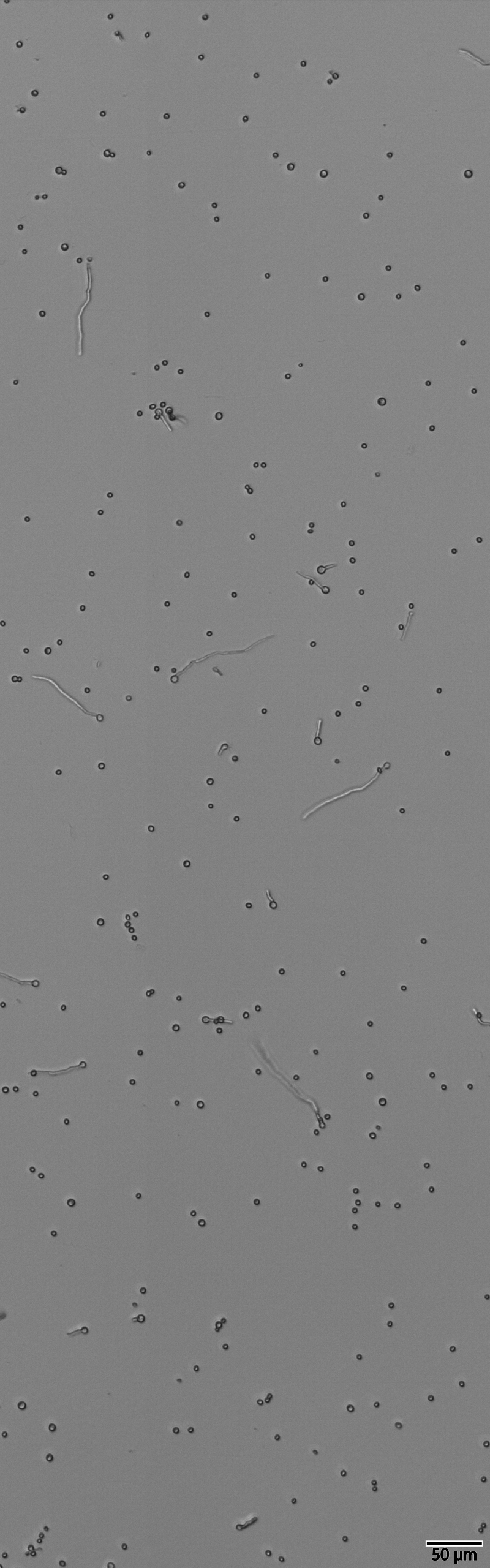


**D**


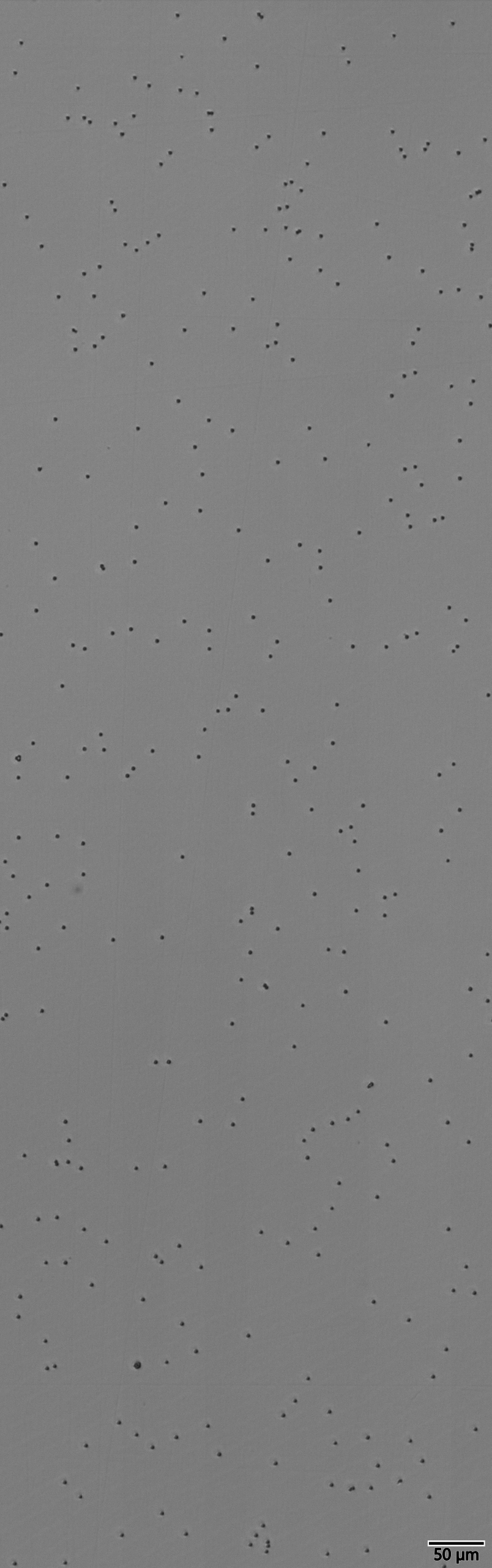


**B**


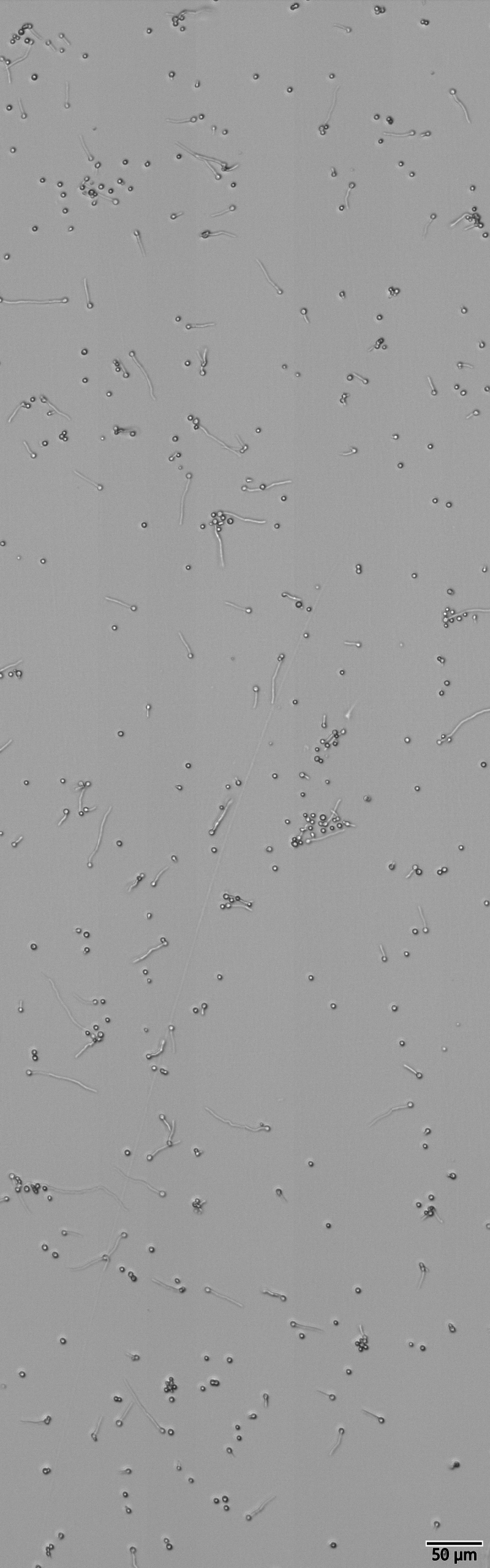


**E**

**Supplemental Figure 1.** Germination of conidia of A. niger (A), A. terreus (B), A. oryzae (C), A. clavatus (D) and A. nidulans (E) in Milli-Q water after 24 h of incubation.


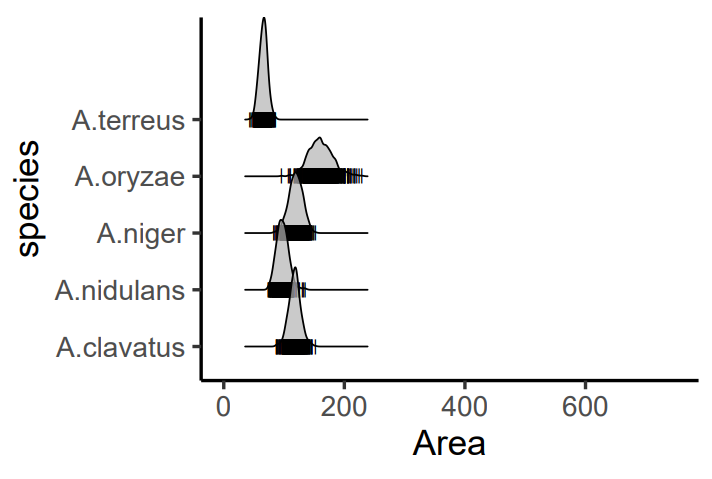

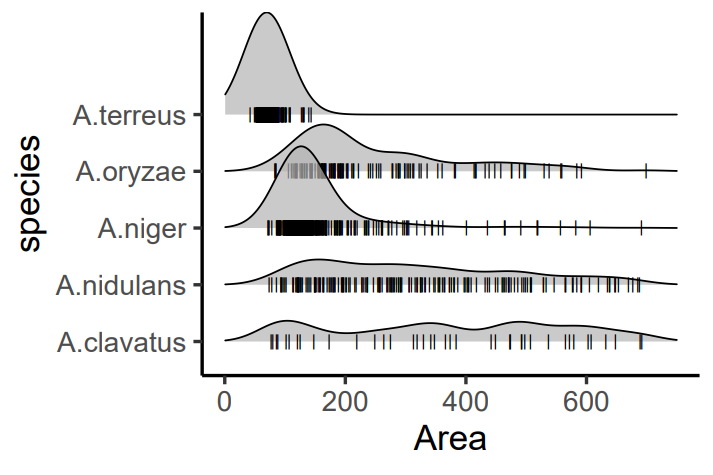

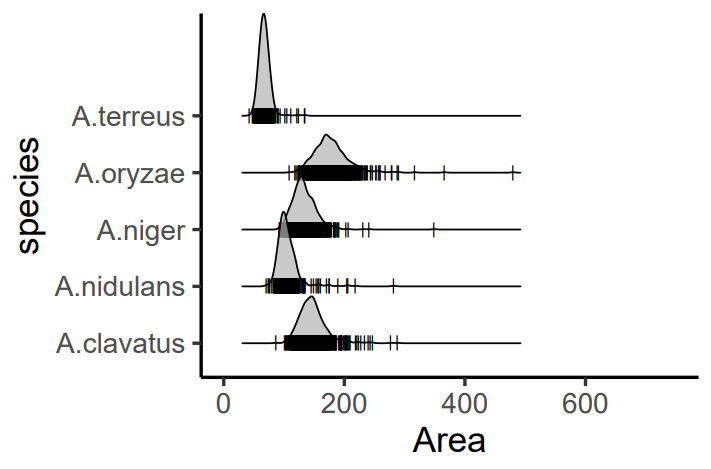


**A**

**B**

**C**

**d = 2.18**

**d = 4.59**

**d = 2.91**

**d = 5.09**

**d = 6.40**

**Supplemental Figure 2.** Normalized density plots of the area of Aspergillus conidia in GNPS at t = 1 h (A), 5 h (B) and 15 h (C). The areas show a variable degree of heterogeneity; a low and high d representing high and low heterogeneity, respectively. Heterogeneity in the swelling response in GNPS at t = 15 h was lowest in the case of A. clavatus with a d of 6.40, while A. terreus showed highest heterogeneity with a d of 2.18 (see Tables 2,3).


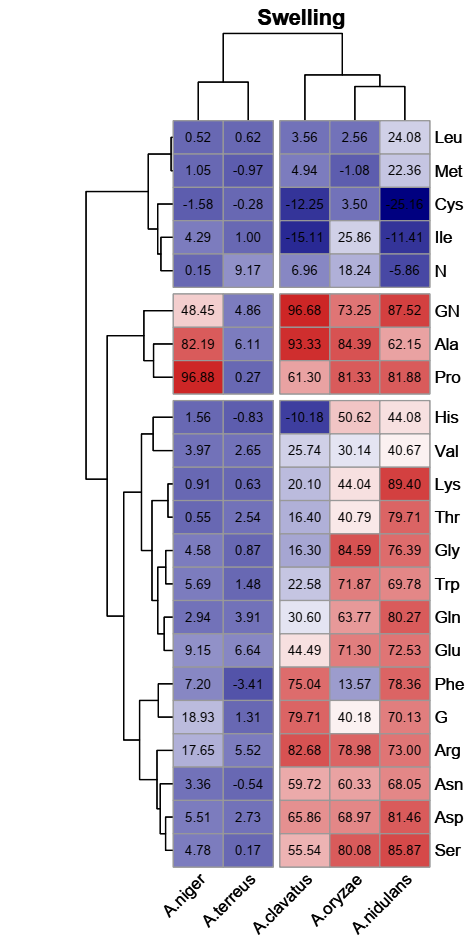

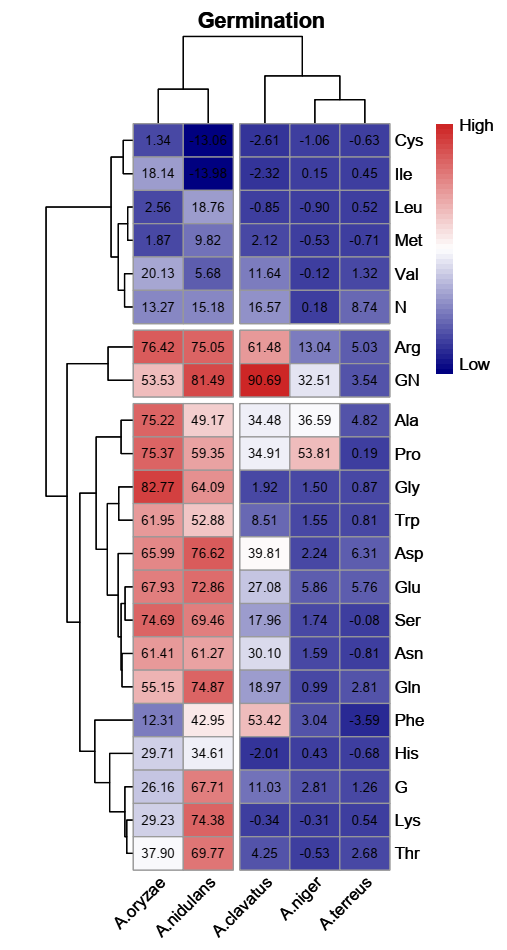


**Germ tube formation**

**Swelling**

**Supplemental Figure 3.** Heat map of normalized P_max_ of swelling and germination of conidia in response to amino acids and glucose (G), nitrate (N) and their combination(GN) in NaPO4 buffer, pH 6, MgSO4 (PS). To this end, the P_max_ of the medium containing additional nutrients (i.e. nitrate, glucose or one of the amino acids) was subtracted from the P_max_ of the PS medium. Tyrosine was excluded from the data set because of the resulting low pH of the medium. Hierarchical clustering shows that low, intermediate and high swelling and germ tube inducing molecules can be distinguished. Moreover, it is shown that clustering of the aspergilli based on swelling and germ tube formation does not follow taxanomy.
